# Bringing Dicynodonts Back to Life: Paleobiology and Anatomy of a New Emydopoid Genus from the Upper Permian of Mozambique

**DOI:** 10.1371/journal.pone.0080974

**Published:** 2013-12-04

**Authors:** Rui Castanhinha, Ricardo Araújo, Luís C. Júnior, Kenneth D. Angielczyk, Gabriel G. Martins, Rui M. S. Martins, Claudine Chaouiya, Felix Beckmann, Fabian Wilde

**Affiliations:** 1 Instituto Gulbenkian de Ciência, Oeiras, Portugal; 2 Museu da Lourinhã, Lourinhã, Portugal; 3 Huffington Department of Earth Sciences, Southern Methodist University, Dallas, Texas, United States of America; 4 Museu Nacional de Geologia, Maputo, Moçambique; 5 Integrative Research Center, Field Museum of Natural History, Chicago, Illinois, United States of America; 6 Centro de Biologia Ambiental, Faculdade de Ciências, Universidade de Lisboa, Lisboa, Portugal; 7 Campus Tecnológico e Nuclear, Instituto Superior Técnico, Bobadela, Portugal; 8 Centro de Investigação em Materiais, Faculdade de Ciências e Tecnologia, Universidade Nova de Lisboa, Caparica, Portugal; 9 Centro de Física Nuclear da Universidade de Lisboa, Lisboa, Portugal; 10 Helmholtz-Zentrum Geesthacht, Geesthacht, Germany; College of the Holy Cross, United States of America

## Abstract

Dicynodontia represent the most diverse tetrapod group during the Late Permian. They survived the Permo-Triassic extinction and are central to understanding Permo-Triassic terrestrial ecosystems. Although extensively studied, several aspects of dicynodont paleobiology such as, neuroanatomy, inner ear morphology and internal cranial anatomy remain obscure. Here we describe a new dicynodont (Therapsida, Anomodontia) from northern Mozambique: *Niassodon mfumukasi* gen. et sp. nov. The holotype ML1620 was collected from the Late Permian K5 formation, Metangula Graben, Niassa Province northern Mozambique, an almost completely unexplored basin and country for vertebrate paleontology. Synchrotron radiation based micro-computed tomography (SRµCT), combined with a phylogenetic analysis, demonstrates a set of characters shared with Emydopoidea. All individual bones were digitally segmented allowing a 3D visualization of each element. In addition, we reconstructed the osseous labyrinth, endocast, cranial nerves and vasculature. The brain is narrow and the cerebellum is broader than the forebrain, resembling the conservative, “reptilian-grade” morphology of other non-mammalian therapsids, but the enlarged paraflocculi occupy the same relative volume as in birds. The orientation of the horizontal semicircular canals indicates a slightly more dorsally tilted head posture than previously assumed in other dicynodonts. In addition, synchrotron data shows a secondary center of ossification in the femur. Thus ML1620 represents, to our knowledge, the oldest fossil evidence of a secondary center of ossification, pushing back the evolutionary origins of this feature. The fact that the specimen represents a new species indicates that the Late Permian tetrapod fauna of east Africa is still incompletely known.

## Introduction

Dicynodonts are an exclusively herbivorous clade of synapsids. They comprise more than 100 species that are known from the Middle Permian the Late Triassic periods Dicynodonts were morphologically disparate and presented a wide range of sizes and putative ecological niches, including semi-aquatic, fossorial, arboreal and grazing [1]. Despite over 150 years of dicynodont research, several paleobiological aspects of the neuroanatomy, inner ear morphology and internal cranium anatomy remain practically obscure [2]–[22]. Moreover, because the destructive technique of serial sampling was the primary way to access the internal anatomy of dicynodont skulls, the data available for various taxa was inconsistent and rarely subjected to synthetic treatments. As a result, details of internal skull anatomy have been overlooked in recent phylogenetic analyses. Only Surkov and Benton [20] included a large number of braincase characters in a phylogenetic analysis of dicynodonts. Recent advances in non-destructive imaging techniques such as high resolution computed tomography, neutron tomography, and synchrotron radiation based micro-computed tomography, hold the potential to provide significant new insight into fossil skull morphology. However, the application of such techniques to dicynodonts has been limited [23]–[27].

**Figure 1:**
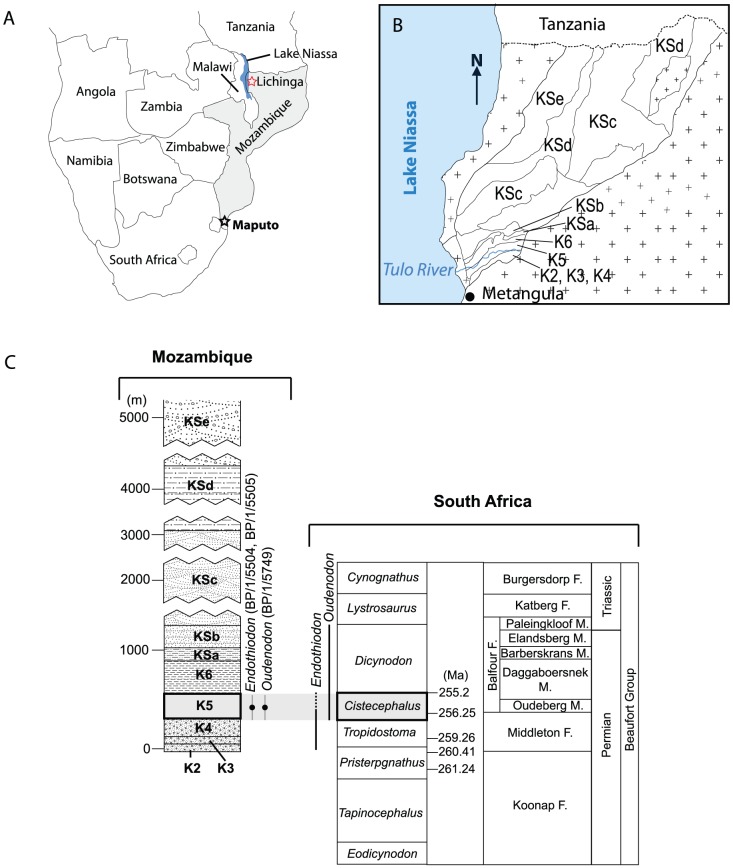
Geological and geographical setting of the fossil site. (A) Fossil site (red star); (B) Geological formation of the Metangula graben (after [30]); (C) Stratigraphic correlation between the Mozambican and South African Karoo (after [30], [138]).

Although located in close proximity to the well-known fossiliferous beds of the Ruhuhu Basin (Tanzania) and the Luangwa Basin (Zambia), the tetrapod fossil record of the Metangula Graben (Mozambique) has received little attention (Fig. 1).

The first discoveries were made by M. Domingos Rocha in 1949 in the context of a geological survey [28]. Subsequent geologic work correlated the Metangula Graben fossiliferous layer to the South African *Tropidostoma* Assemblage Zone [29]–[31]. The fossils collected in 1949, along with additional specimens collected in 1954, were sent to S. Henry Haughton in South Africa, who briefly referred to them in a broader article [31]. Later, M. Telles Antunes described in detail several skull elements of *Endothiodon* and an unidentified gorgonopsian from the Niassa province [32]. Some years after, Latimer at al. [33] examined patterns of tooth replacement in *Endothiodon* using some of the material initially reported by Haughton.

**Figure 2:**
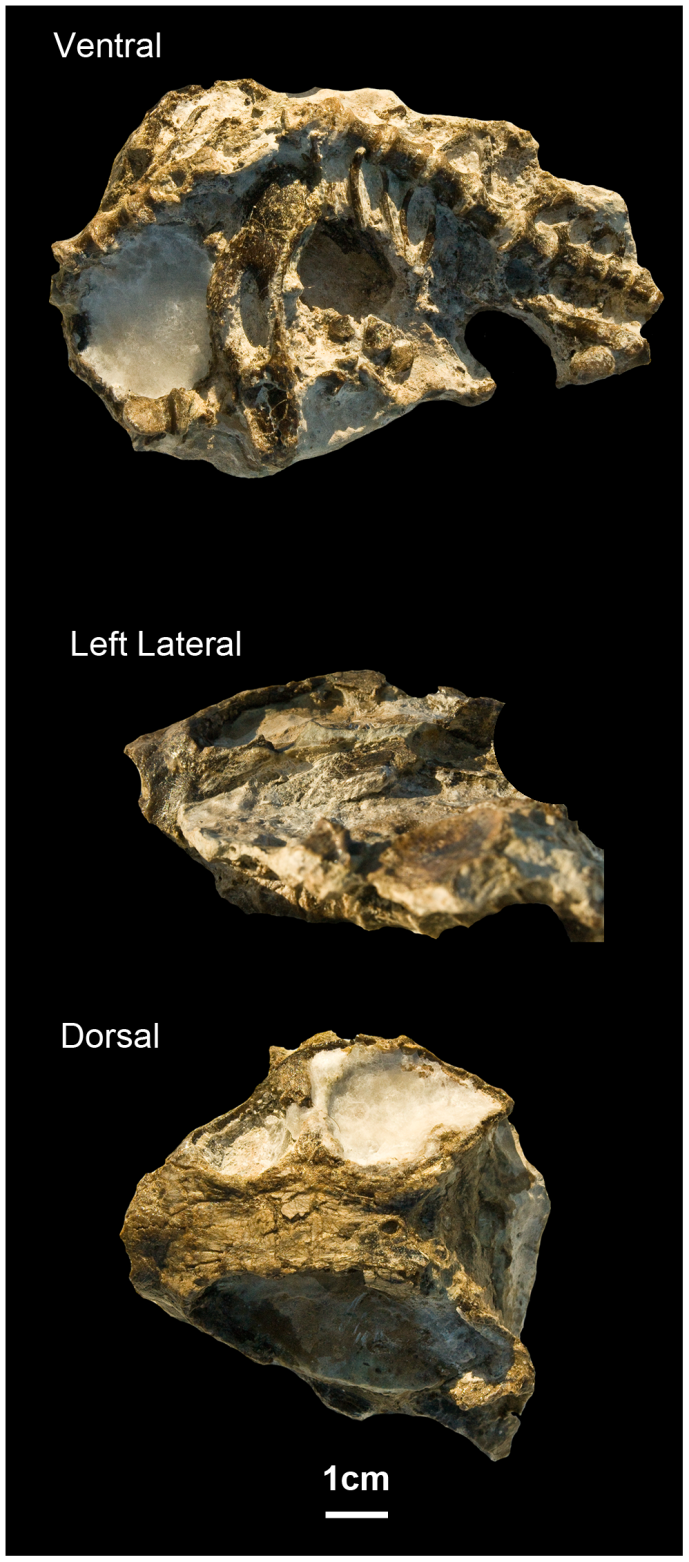
ML1620 (*Niassodon mfumukasi* holotype) in ventral, dorsal and left lateral views.

In July of 2009, our team began new paleontological fieldwork in the Metangula Graben, in which we relocated the historical fossil localities [32] and found new ones [28]. Among the material collected during this 2009 expedition, under the auspices of Projecto PalNiassa, is a small dicynodont specimen (ML1620) that is the focus of this paper (Fig. 2). Despite other sporadic paleontological studies [32], [34]–[36], Projecto PalNiassa represents the only systematic long-term paleontological project ever conducted in Mozambique.

Here, we describe the anatomy of a new dicynodont genus and species from northern Mozambique, investigate its phylogenetic relationships and biostratigraphic implications, and assess its ontogenetic stage based on gross morphology and bone histology. As part of this work, we present the first fully segmented 3D model of a dicynodont skull and mandible derived from high-resolution micro-computed tomography (µCT) data (Figure S1). The model and associated digital endocast provide novel information on the ethmoid, parasphenoid, and prootic, as well as new insights on the osseous labyrinth, cranial nerves, cranial vasculature, and brain anatomy.

## Methods

### Ethics Statement

The fossil described here (ML1620) was collected under the auspices of the Projecto PalNiassa (www.palniassa.org) under an institutional protocol between the Museu Nacional de Geologia (Maputo, Mozambique) and the Grupo de Etnologia e Arqueologia da Lourinhã - Museu da Lourinhã (Lourinhã, Portugal). All specimens collected under the terms of this protocol belong to the Republic of Mozambique and are studied under explicit authorization of Museu Nacional de Geologia (Ministério dos Recursos Minerais, Maputo, Mozambique). ML1620 is presently housed at the Museu da Lourinhã and will return to Mozambique. All necessary permits were obtained for this study, which complied with all relevant regulations.

### Nomenclatural Acts

The electronic edition of this article conforms to the requirements of the amended International Code of Zoological Nomenclature (ICZN), and hence the new names contained herein are available under that Code from the electronic edition of this article. This published work and the nomenclatural acts it contains have been registered in ZooBank, the online registration system for the ICZN. The ZooBank LSIDs (Life Science Identifiers) can be resolved and the associated information viewed through any standard web browser by appending the LSID to the prefix “http://zoobank.org/”. The LSID for this publication is: urn:lsid:zoobank.org:pub:2C5D6A8B-3BA4-44EE-A5F0-E7E1075D82B6. The electronic edition of this work was published in a journal with an ISSN, and has been archived and is available from the following digital repositories: PubMed Central (http://www.pubmedcentral.nih.gov/), LOCKSS (http://www.lockss.org/lockss/).

### Preparation

The specimen was prepared using both mechanical and chemical techniques. For mechanical preparation, matrix was removed using a PaleoTools Microjack #4 and exposed bone was strengthened by impregnation with polyvinyl acetate. Chemical preparation was done by alternation of 1–2 hour baths in 2–4% formic acid buffered with calcium phosphate and several hours in water. This technique is similar to that one used by Latimer et al. [33] on the fossils they examined from the Metangula Graben.

### X-ray tube-based high-resolution computed tomography (µCT) and 3D reconstruction

We performed µCT imaging with a phoenix nanotom® s scanner (phoenix, GE Measurement & Control Solutions, Germany) equipped with a 180 kV/15 W high-power nanofocus® tube. The specimen was scanned using tube operation mode 0 (High Power) with an acceleration voltage of 160 keV and e-beam current of 20 µA. A total of 1200 projections were acquired for a full revolution. Due to scanning volume restrictions, two scans were performed on two different regions of the fossil in order to cover its whole volume. The tomograms were reconstructed from the 2D projections using datos|x2.0 reconstruction software (phoenix, GE Measurement & Control Solutions, Germany) resulting in a voxel size of 30 µm. The two scans were stitched into a single tomogram representing the whole fossil using the FIJI (Fiji Is Just ImageJ) software and the 3D stitching plugin [37], [38]. To facilitate data processing and handling the tomogram was downsampled to a 60-µm resolution and cropped using the FIJI software. Further data processing included (i) reorientation and re-sectioning of the volume to obtain axial sections in orthogonal anatomical orientation, (ii) manual segmentation of different bones (iii) surface reconstruction of the individual segmented bones, and (iv) repositioning of the cranium, mandible and axial skeleton to anatomical relevant positions, all done with the Amira V5.3 software (Visualization Sciences Group, France). The surface renderings of the individual bones were then converted to Wavefront “obj” files, and assembled again to a 3D pdf model (Figure S1) using the SimLab software.

Given the bone-to-bone contact matrix in ML1620 (Table S1), we searched for a color code maximizing the color difference between adjacent bones.

### Synchrotron Radiation Based Micro-Computed Tomography (SRµCT)

**Figure 3:**
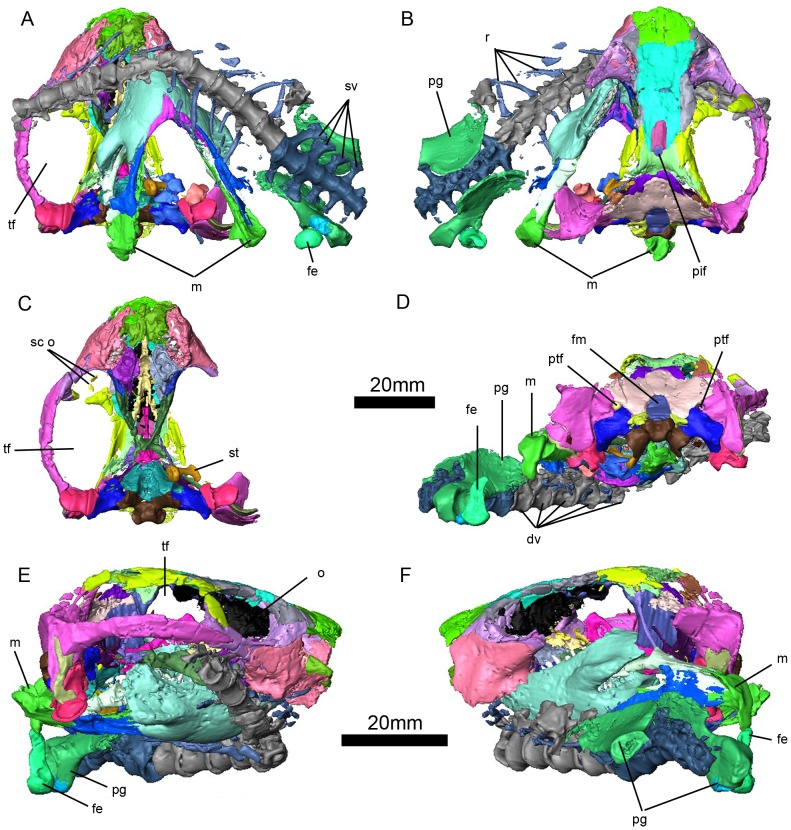
*Niassodon mfumukasi* 3D rendering, after segmentation of individual bones according to bone color code .(see methods section). Ventral (A); dorsal (B); skull in ventral (C); posterior (D); lateral right (E); lateral left (F) views. **dv**, dorsal vertebrae; **fe**, femur; **fm**, foramen magnum; **o**, orbit; **m**, mandible; **pg**, pelvic girdle; **pif**, pineal foramen; **ptf**, post temporal fenestra; **r**, ribs; **sco**, sclerotic ossicles; **st**, stapes; **sv**, sacral vertebrae; **tf**, temporal fenestra.

**Figure 4:**
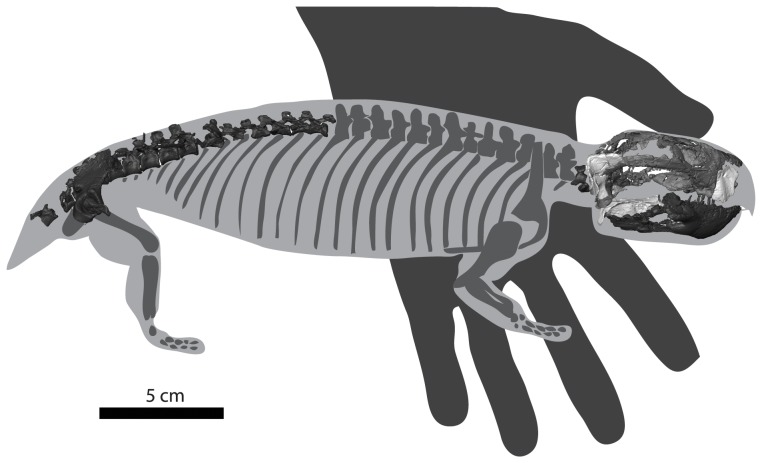
Preserved skeletal elements of Niassodon mfumukasi (ML1620) imposed on a *Pristerodon* silhouette, adapted from [146]. Skeletal parts repositioned are in dark grey. Skeletal parts mirrored from the other side are in light grey. Skeletal parts in the original position are in intermediate grey.

**Figure 5:**
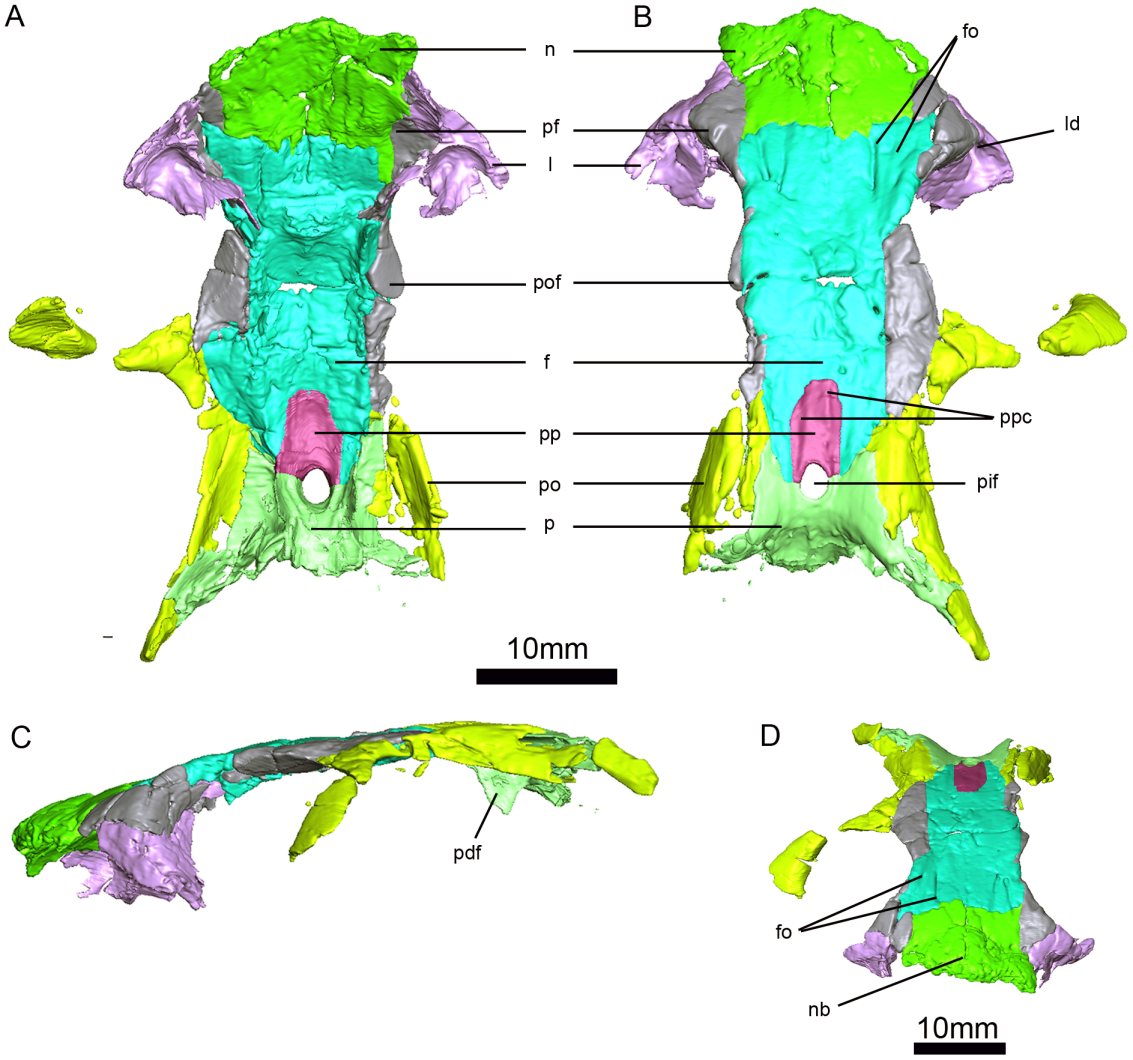
*Niassodon mfumukasi* skull roof. Ventral (A), dorsal (B), left lateral (C), anterodorsal (D) views. **f**, frontal; **fo**, frontal ornamentation; **l**, lacrimal; **ld**, lacrimal duct; **n**, nasal; **nb**, nasal boss; **p**, parietal; **pdf**, descending flange of the parietal; **pf**, prefrontal; **pif**, pineal foramen; **po**, postorbital; **pof**, postfrontal; **pp**, preparietal; **ppc**, preparietal crests.

**Figure 6:**
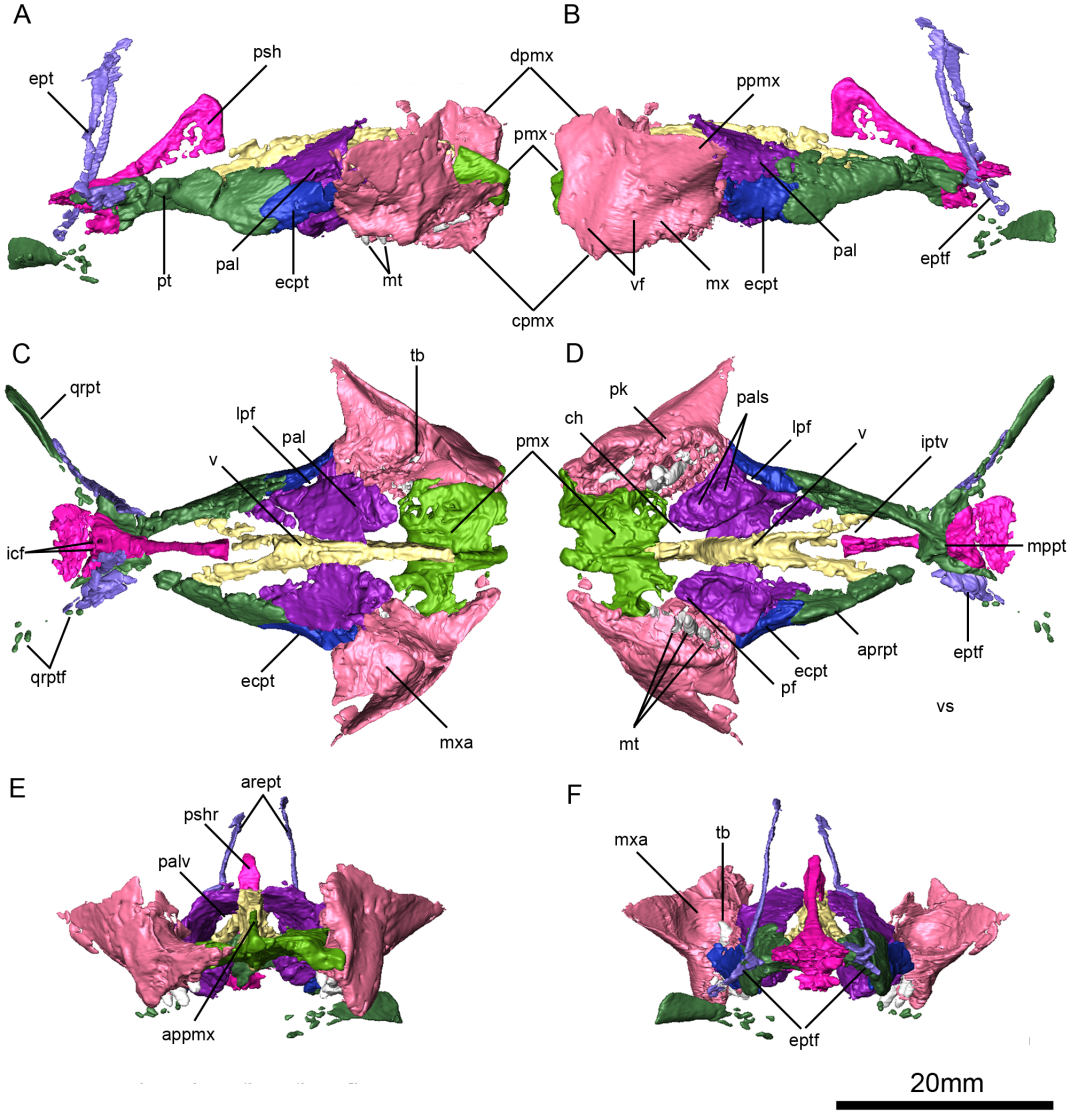
*Niassodon mfumukasi* palate. Left lateral (A), right lateral (B), ventral (C), dorsal (D), anterior (E), posterior (F) views. **appmx**, ascending process of the premaxilla; **aprpt**, anterior palatal ramus of the pterygoid; **arept**, ascending ramus of the epipterygoid; **ch**, choanae; **cpmx**, caniniform process of the maxilla; **dpmx**, dorsal process of the maxilla; **ecpt**, ectopterygoid; **ept**, epipterygoid; **eptf**, epipterygoid foot; **icf**, internal carotid foramen; **iptv**, interpterygoid vacuity; **lpf**, lateral palatal foramen; **mppt**, medium plate of the pterygoid; **mt**, maxillary teeth; **mx**, maxilla; **mxa**, maxillary antrum; **pal**, palatine; **pals**, palatine sulci; **palv**, palatine vacuity; **pf**, palatal foramen; **pk**, postcaniniform keel; **pmx**, premaxilla; **ppmx**, posterior process of the maxilla; **psh**, parashenoid; **pshr**, parashenoid rostrum; **pt**, pterygoid; **qrpt**, quadrate ramus of pterygoid; **qrptf**, quadrate ramus of pterygoid fragments; **tb**, tooth bub; **v**, vomer; **vf**, vascular foramina.

**Figure 7:**
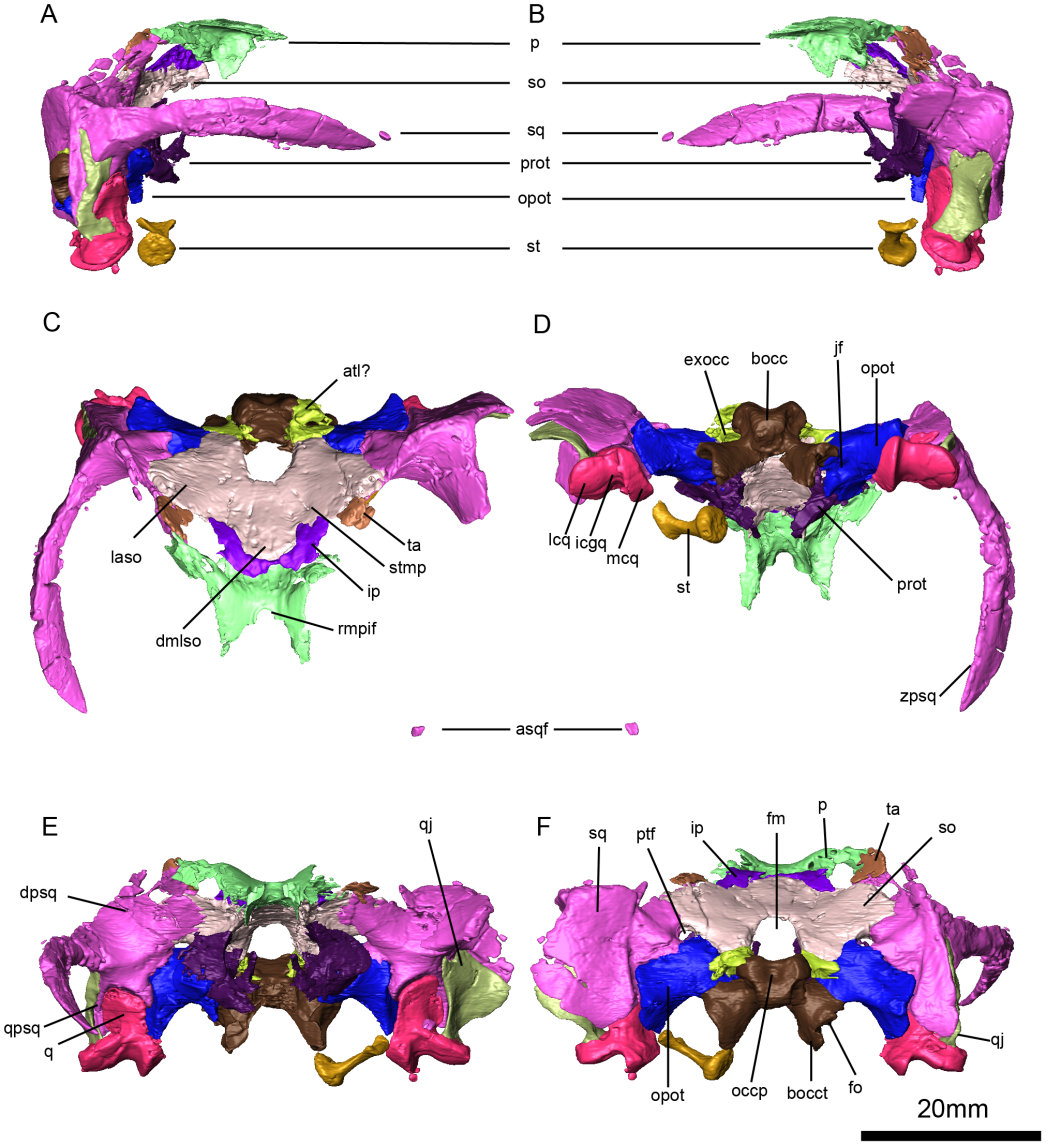
*Niassodon mfumukasi* occipital region. Right lateral (A), left lateral (B), dorsal (C), ventral (D), posterior (E), anterior (F) views. **asqf**, anterior left squamosal fragment; **atl**, parcial atalantal ring; **bocc**, basioccipital; **dmlso**, dorsal median lobe of the supraoccipital; **dpsq**, dorsal process of the squamosal; **exocc**, exoccipital; **fm**, foramen magnum; **fo**, fenestra ovalis; **jf**, jugular foramen; **icgq**, intercondylar groove of the quadrate; **ip**, interparietal (postparietal); **laso**, lateral ala of the supraoccipital; **lqc**, lateral condyle of the quadrate; **mcq**, medial condyle of the quadrate; **occp**, occipital pit; **opot**, opisthotic; **p**, parietal; **prot**, prootic; **ptf**, postemporal fenestra; **q**, quadrate; **qj**, quadratojugal; **qpsq**, quadrate process of the squamosal; **so**, supraoccipital; **sq**, squamosal; **st**, stapes; **zpsq**, zigomatic process of the squamosal.

**Figure 8:**
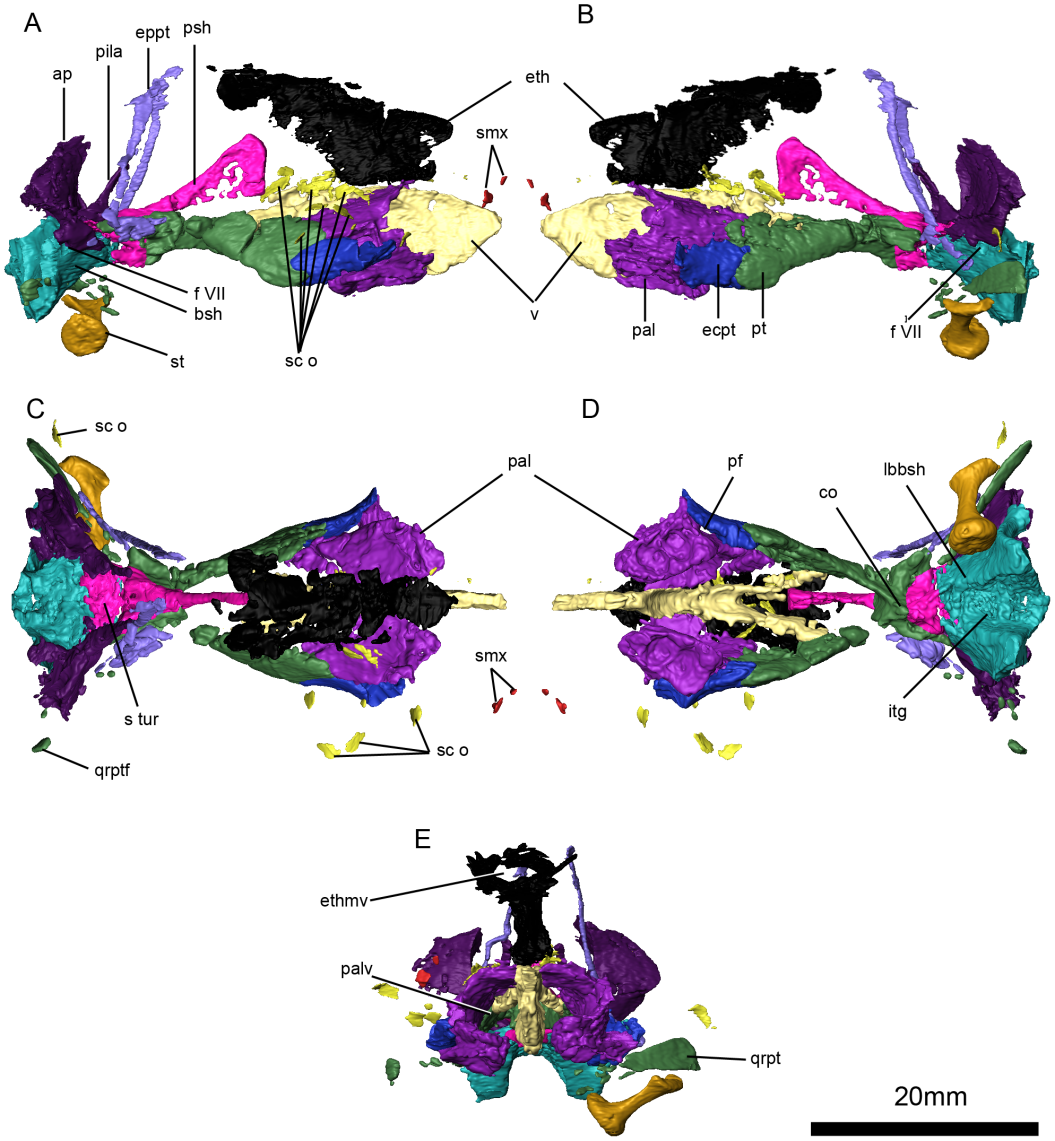
*Niassodon mfumukasi* internal cranial bones. Right lateral (A), left lateral (B), dorsal (C), ventral (D), and anterior (E) view. **ap**, alar process of the prootic, **bsh**, basisphenoid; **co**, crista osophagea; **ecpt**, ectopterygoid; **eppt**, epipterygoid; **eth**, ethmoid; **ethmv**, ethmoid vacuity; **f VII**, facial foramen; **itg**, intertuberal groove; **lb**, lateral buttress; **pal**, palatines; **palv**, palatine vacuity; **pal** **f**, palatal foramen; **pila**, pila antotica of the prootic; **psh**, parasphenoid; **pt**, pterygoid; **qrpt**, quadrate ramus of the pterygoid; **qrptf**, quadrate ramus of the pterygoid fragments; **s** **tur**, sella turcica; **sc** **o**, sclerotic ossicles; **smx**, septomaxilla; **v**, vomer.

**Figure 9:**
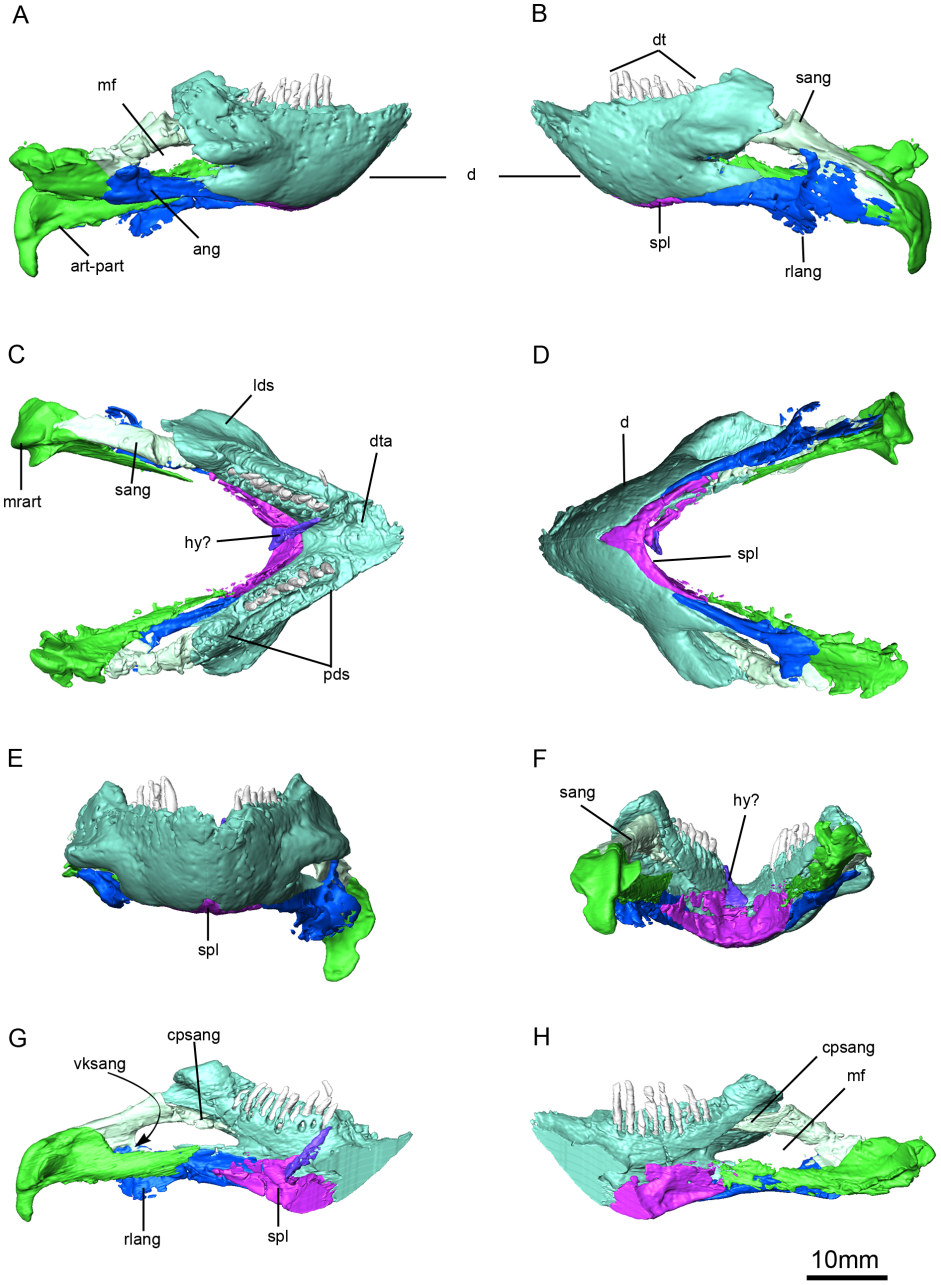
*Niassodon mfumukasi* mandible. Left lateral (A), right lateral (B), anterior (C), posterior (D), ventral (E), dorsal (F) views; right ramus in medial view (G) and left ramus in medial view (H). **ang**, angular; **art-part**, articular-prearticular complex; **cpsang**, conical process of the surangular; **dt**, dentary teeth; **dta**, dentary table; **hy?**, probable hyoid; **mrart**, median ridge of the articular; **lds**, lateral dentary shelf; **pds**, postdentary sulcus; **rlang**, reflected lamina of the angular; **sang**, surangular; **spl**, splenial; **vksang**,ventral keel of the surangular.

**Figure 10:**
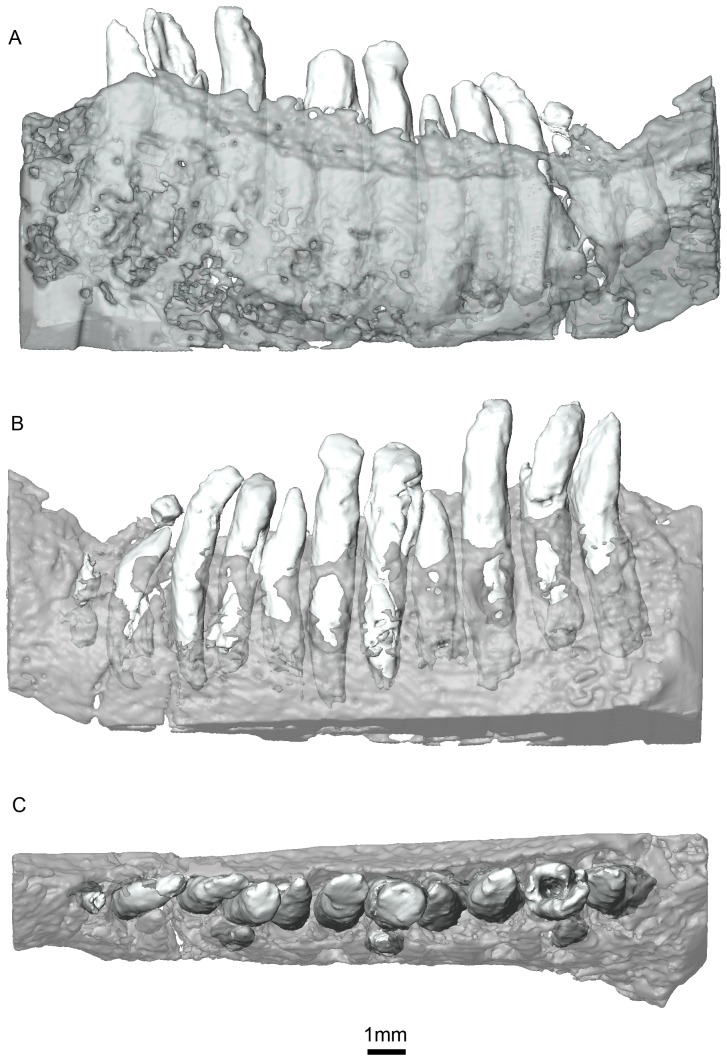
*Niassodon mfumukasi* left dentary. teeth in lateral (A), medial (B) and, dorsal (C) views. Part of the dentary was erased medially so that the teeth implantation can be seen. Note the presence of three replacement teeth medially located to the functional tooth row.

**Figure 11:**
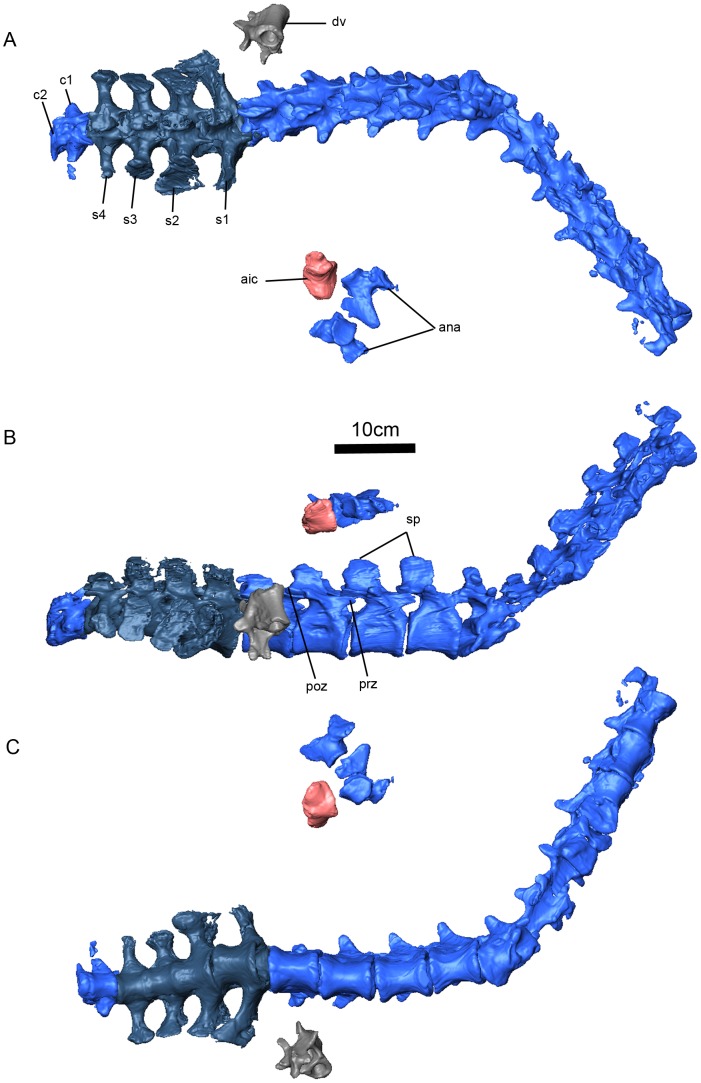
*Niassodon mfumukasi* vertebral column. Dorsal (A), right lateral (B), ventral (C) views. **ana**, atlas neural arches; **aic**, atlas intercentrum; **c**, caudal vertebra; **dv**, dorsal vertebra; **poz**, postzygapophysis; **prz**, prezygapophysis; **s**, sacral vertebra; **sp**, spinous processes.

**Figure 12:**
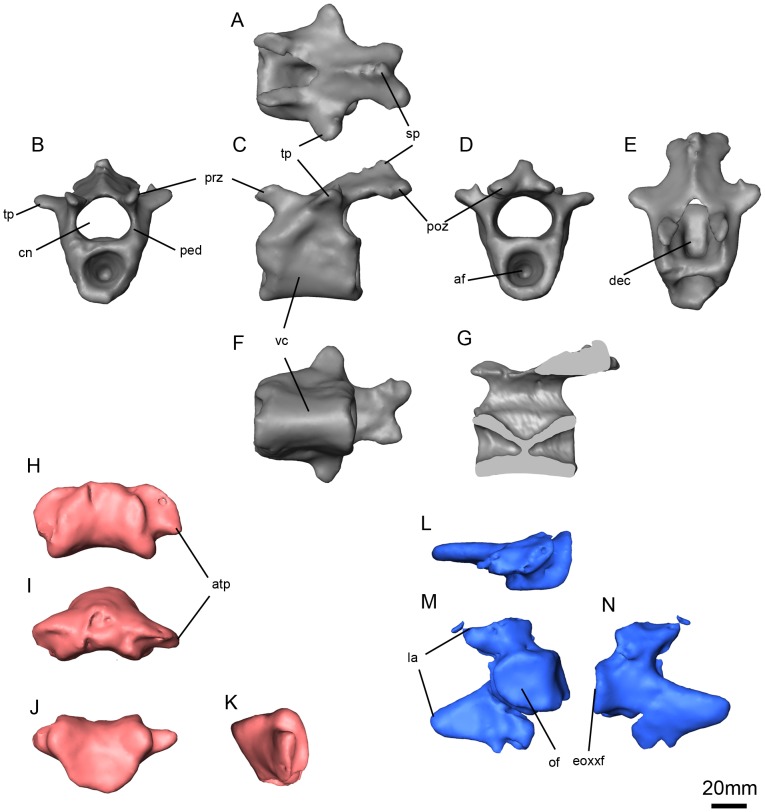
*Niassodon mfumukasi* caudal vertebra and atlas. Caudal vertebra in dorsal (A), anterior (B), left lateral (C), posterior (D), anterodorsal (E), ventral (F) views and sagittal section (G); atlas intercentrum in ventral (H), anterior (I), dorsal (J), right lateral (K) views; left atlas neural arch in dorsal (L), medial (M), left lateral (N) views. **af**, articular facet; **atp**, atlas intercentrum transverse process; **cn**, neural canal; **dec**, dorsal excavation on the centrum; **exoccf**, exoccipital facet; **la**, lateral.

**Figure 13:**
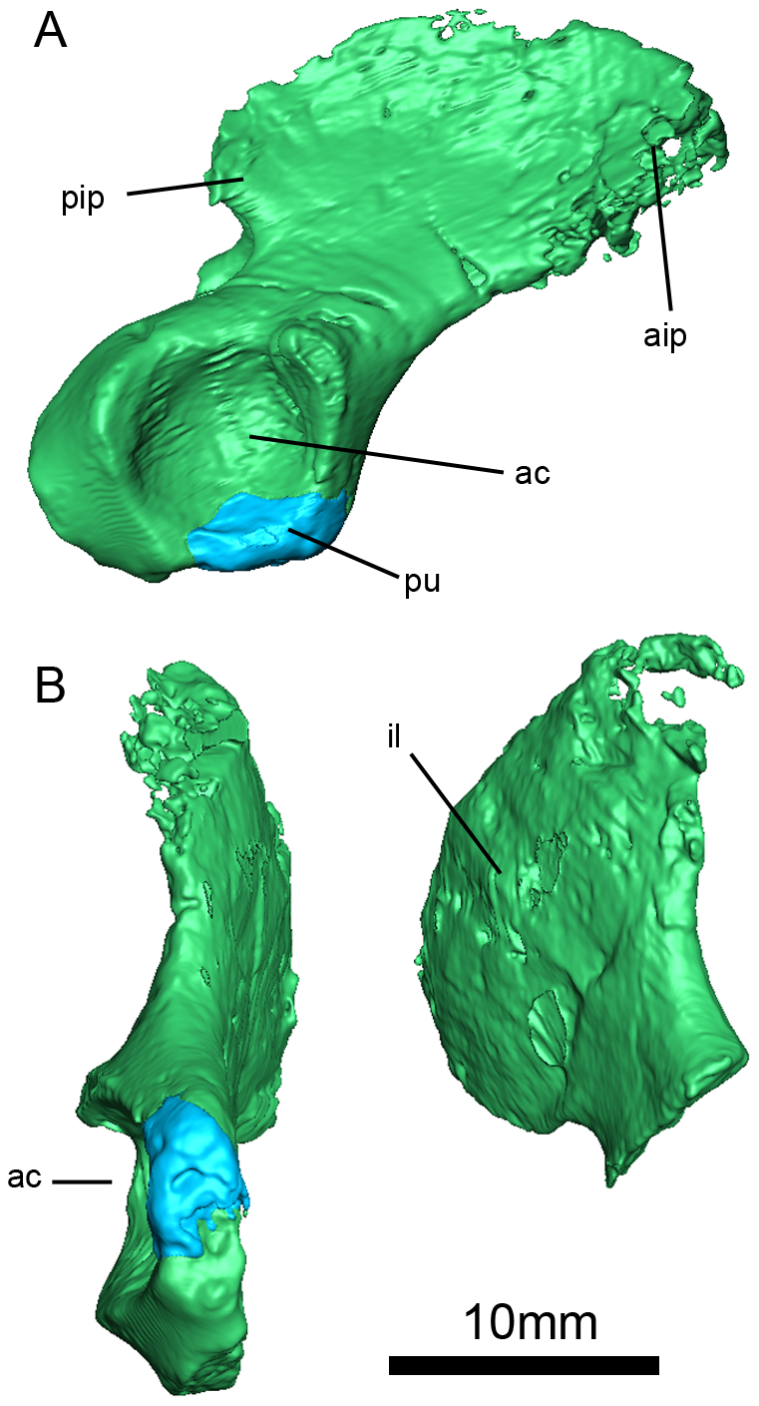
*Niassodon mfumukasi* pelvic girdle. pip , posterior iliac process; **aip**, anterior iliac process; **ac**, acetabulum; **pu**, pubis; **il**, ilium.

**Figure 14:**
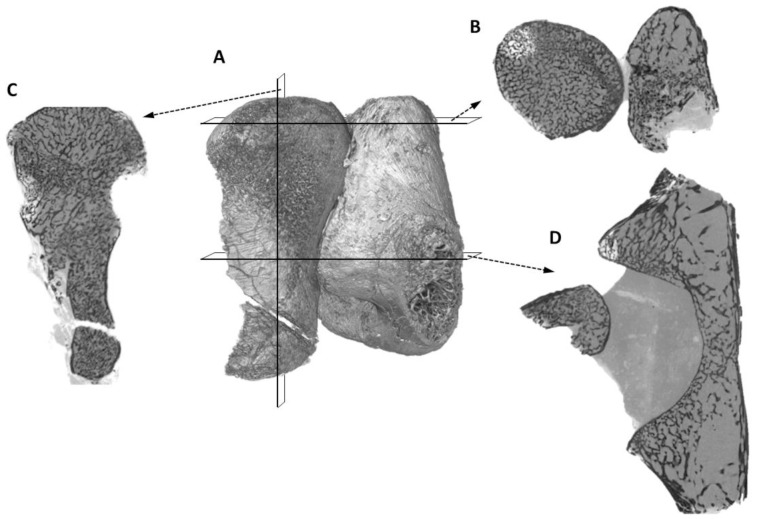
SR∀CT virtual histological sections of the femur and partial pelvic girdle of *Niassodon mfumukasi*. (A) Volume rendering of the femur and partial pelvic girdle; (B) Proximal epiphyseal section of the femur; (C) longitudinal section along the femur; (D) diaphyseal section of the femur and acetabulum (note that the femur is not in anatomical position, but as it was found in the fossil, i.e., the femur epiphysis is dislodged from the acetabulum).

To investigate internal bone morphology, a fragment that included the femur and pelvic girdle was scanned using the SRµCT facility at the HARWI II beamline operated by the Helmholtz-Zentrum Geesthacht at the storage ring DORIS III at the Deutsches Elektronen–Synchrotron in Hamburg, Germany. Technical details of the beamline used are described in Beckmann et al. [39], [40], Reimers et al. [41] and Herzen [42]. We imaged the sample in absorption mode with photon energy of 60 keV. We acquired 900 projections within 180°. The tomographic reconstruction was performed using an implementation of “back-projection of filtered projections” [43]; the effective final pixel size was 18.5 µm. We used VGStudio Max 2.1 (Volume Graphics, Heidelberg, Germany) to perform the initial visualization. With the exception of the external anatomical traits, all internal measurements were made with VGStudio Max 2.1. The 3D reconstructions presented in Fig.3, 4, 5, 6, 7, 8, 9, 10, 11, 12, 13, 14, 15 were done using Amira V.5.3.

**Figure 15:**
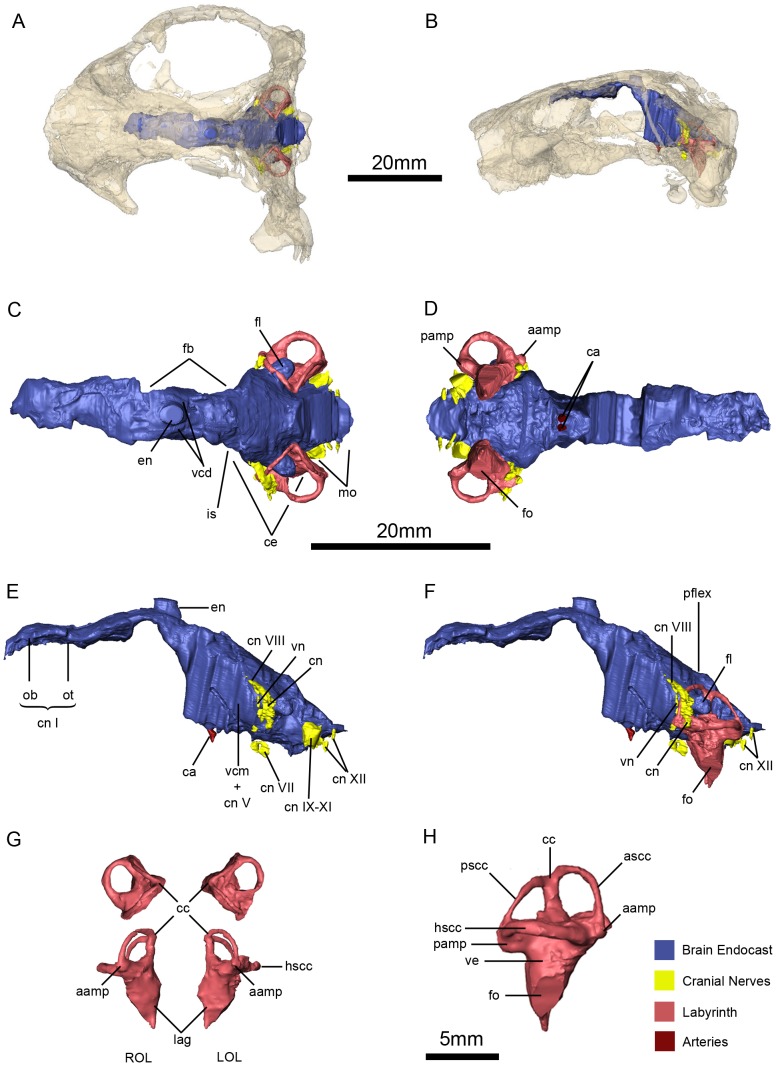
*Niassodon mfumukasi* neuroanatomy and inner ear anatomy. Cranial endocasts in the cranium in dorsal (A), and left lateral (B) views. Cranial endocasts in dorsal (C), ventral (D), left lateral without the osseous labyrinth (E), left lateral view with the osseous labyrinth (F). Both osseous labyrinths in dorsal and anterior views (G) and both osseous labyrinths in right lateral view (H). **aamp**, ampulla of the anterior semicircular canal **ca**, carotid arteries; **ascc**, anterior semicircular canal; **cc**, crus comunis; **ce**, cerebelum; **cn**, cochlear nerve; **cn** **I,** olfactory nerve; **cn** **VII**, facial nerve; **cn** **VIII**, vestibulocochlear nerve; **cn** **IX-XI**, glossopharyngeal and vagoaccessory nerves; **cn** **XII**, hypoglossal nerves; **en**, epiphyseal nerve; **fb**, forebrain; **fl**, paraflocculus; **is**, isthmus; **hscc**, horizontal semicircular canal; **hy**, hypophysis; **LOL**, left osseous labyrinth; **mo**, medula oblongata; **lag**, lagena; **ob**, olfactory bulb; **ot**, olfactory tract; **pamp**, ampulla of the posterior semicircular canal; **pflex**, pontine flexure **pscc**, posterior semicircular canal; **ROL**, right osseous labyrinth; **vcm +** **cn** **V**, vena capitis medialis and trigeminal nerve; **vcd**, vena capitis dorsalis; **vn**, vestibular nerve.

Although, in some cases, the exact measures of the neuroanatomy cannot be inferred based exclusively on the volumes of the cranial endocast [44], [45] we assume that endocranial volume does provide an approximate and reliable estimate of brain size [46]. Therefore we refer to the different identifiable volumes by their neuroanatomical names (e.g. brain, inner ear, cranial nerves). One of the reasons why the delimitation of the brain is difficult in non-mammalian synapsids is because the ethmoid is usually unossified, making the anterior boundaries of the brain speculative [17]. In ML1620, the ethmoid region is preserved in great detail, allowing the reconstruction of the endocranial cavity with unprecedented accuracy, even though the anterior forebrain boundary is not delimited by any osseous structure. The brain is delimited by the basioccipital, exoccipital, supraoccipital and opisthotic posteriorly; by the postparietal, parietal, preparietal, and frontal dorsally; prootic and epipterygoid laterally; parasphenoid and basisphenoid ventrally; and the olfactory tracts and bulbs are surrounded by the ethmoid. The only region that is not delimited by bone is the ventral border of the cerebrum (see Neuroanatomy and Inner Ear Morphology section below), and the reconstruction presented here represents the minimum volume for that portion of the brain.

### Bone Color Code

In order to distinguish all segmented structures (bones and soft tissues) in the 3D rendering, each needs a distinctive color. Given that the process simply consists of assigning colors to specified volumes, this can be done without any logical criterion and indeed there is no standard color code for cranial bones. Therefore, different publications use different codes, and the codes adopted can even change between papers from the same authors. Here, considering a convenient RGB color ramp, we used an algorithm that tries to maximize the color differences between adjacent bones. The obtained code could be used as a standard for cranial bone colors. However, this code can be improved by relying on a well-founded color distance in the space of human color perception, and by performing a local search procedure combined with the construction strategy suggested in Resende et al [47]. To develop a truly universal and standardized set of colors that would apply to other clades with different numbers and arrangements of elements in the skull, a significant number of skulls should be compiled in a single matrix. Such endeavor is beyond the scope of this paper.

Given the bone-to-bone contact matrix in ML1620 (Table S1), we searched for a color code maximizing the color difference between adjacent bones. This optimization problem is related to the antibandwidth problem, a Non-deterministic Polynomial-time hard problem that still motivates the development of heuristics to generate sub-optimal solutions. Here, we relied on a construction strategy proposed by Resende et al. [47] to obtain a satisfactory bone color code (Table S1) that was used to color the segmented bones (Fig. 3, 5, 6, 7, 8, 9, 11, 12, 13, 15; Fig. S1).

### Phylogeny

To investigate the phylogenetic relationships of the specimen, we added it to the data matrix of Kammerer et al. [48] (Text S1). The final data set includes 174 characters. From these characters, 153 are discrete binary or multistate characters, and we treated these characters as unordered and of equal weight. The remaining 21 characters are continuous. To code the continuous characters, we added a small number of new measurements to the database of Kammerer et al. [48]. The data matrix can be found in the Table S1. We treated the continuous characters as additive [49], and used mean values as coding for the operational taxonomic units (OTUs) except in cases when only a single measurement was available for an OTU. We coded unknown and inapplicable discrete state and continuous characters as ‘?’ [50]. We analyzed the data set using TNT 1.1 (October 2010 version) [51], and we employed two search strategies. For the first search, we used the new technology methods of TNT. We performed a driven search with the initial search level set at 65, which was checked every three hits. The initial number of addition sequence replicates was 500, and we required the search to find the trees of shortest length 20 times. We started the analysis with default settings for sectorial searching, tree drifting, parsimony ratchet, and tree fusing. In the second analysis, we used the traditional search method of tree bisection and reconnection (TBR) branch swapping with 10,000 replicates, with 10 trees held per replicate. We used *Biarmosuchus* to root the most parsimonious cladograms from both analyses. To measure support for the most parsimonious cladograms, we utilized symmetric resampling [52] and decay analysis [53], [54]. Our symmetric resampling results are based on 15,000 replicates, with 10 replicates of TBR branch swapping with two trees held per replicate for each resampling replicate. The decay analysis results are based on a sample of 463,436 suboptimal cladograms with lengths up to seven steps longer than the most parsimonious cladograms. Following the previous recommendations [51], we generated the suboptimal trees through a series of traditional searches in which we incrementally increased the length of suboptimal cladograms retained as well as the number of suboptimal cladograms. The resulting cladograms were filtered to remove duplicates before the decay analysis, so the 463,436 cladograms in the sample are all unique.

### Body size estimation

Because patterns of limb scaling have not been examined in detail among dicynodonts, and postcranial material has not been identified for a number of otherwise well-known dicynodont taxa, basal skull length is sometimes used as a proxy for body size in the clade (e.g. [55], [56]). The snout of ML1620 is damaged, preventing a direct measurement of basal skull length. To estimate the approximate value for this measurement, we assumed that ML1620 had comparable proportions to CGP/1/2215, a nearly complete skull that likely can be referred to *Cryptocynodon simus*. Although CGP/1/2215 is larger than ML1620, the specimens show some similarities, such as the presence of relatively long tooth rows, the shape of the palatines, and the relatively posterior location of the pineal foramen, that make it possible to align the specimens closely. Based on this alignment and the assumption of similar proportions, we estimate that the original basal skull length of *Niassodon* was approximately 63 mm.

Body mass estimates for nonmammalian synapsids are relatively rare in the literature [57]–[60]. An estimation of the mass of ML1620 was needed that did not required the use of long bone lengths since humeri are not preserved in ML1620, and only the proximal end of one femur is present. This precludes the direct use of humerus or femur dimensions in allometric scaling equations. However, it was recently shown that skull length was highly correlated with femur length in a small, but phylogenetically wide-ranging sample of synapsids [61], and they used the resulting scaling equation to estimate femur lengths for use in their study of trends in body size. We used a similar approach to estimate humerus and femur lengths for ML1620, and subsequently used those values to estimate body mass (see methods and Table S2). To predict the length of the humerus of ML1620, we assembled a dataset of 46 anomodont specimens with associated skulls and humeri that we have personally examined (Table S2). Because our measurements of skull length and humerus length include some error, we used reduced major axis regression (RMA) [62] to model the relationship between skull length and humerus length. The results of this analysis show that these values are highly correlated, and the RMA model fits the data very well (r^2^ = 0.924; p<<0.001). We used the equation for the RMA regression line and our estimated skull length (63 mm) to derive an estimated humerus length of approximately 17 mm. Our femur length estimates are based on a similar general procedure, but we used three different datasets in the process. The first consisted of a dataset of 26 anomodont specimens with associated skulls and femora that we have personally examined, and which we subjected to RMA regression (Table S2). The second data set included these 26 specimens as well as values for six additional specimens taken from previously published databases [61]. The RMA models fit these data very well (r^2^ = 0.903, p<<0.001; r^2^ = 0.909, p<<0.001, respectively), and we used the equations for the RMA regression lines and our estimate skull length to derive predicted femur lengths. We derived our third femur length estimate by using our estimated skull length of ML1620 in the synapsid-wide regression equation [61]. The femur lengths predicted from these three datasets ranged from approximately 40 mm to approximately 54 mm.

Campione and Evans [63] recently presented new regression equations for estimating tetrapod body mass and evaluated the error associated with the use of various limb dimensions as the basis for these predictions. They found that humerus and femur circumference generally gave superior results compared to using humerus and femur length, but because we did not have circumference data available in our analyses, we employed their equations that use humerus and femur length. We derived a total of seven body mass estimates using our humerus and femur lengths. The first of these was based on our predicted humerus length and used the non-phylogenetically corrected humerus length equation [63]. The second through fourth estimates were based on our three predicted femur lengths and were calculated using the non-phylogenetically corrected femur length equation [63]. The final three estimates were based on the non-phylogenetically corrected multivariate equation for humerus and femur length [63], using our predicted humerus length and each of our three predicted femur lengths. Our predicted body masses for *Niassodon* ranged from approximately 364g to approximately 849g (mean 491g; see Fig. 4).

### SYSTEMATIC PALEONTOLOGY

SYNAPSIDA Osborn, 1903

THERAPSIDA Broom, 1905b

ANOMODONTIA Owen, 1859

DICYNODONTIA Owen, 1859

EMYDOPOIDEA (van Hoepen, 1934)

KINGORIIDAE King 1988 *sensu* Kammerer and Angielczyk 2009

*Niassodon* n. gen.

urn:lsid:zoobank.org:act:EA01AA88-D90B-40FB-89ED-DBDF4A33B548

### Etymology

Niassodon is a composite of the two words Niassa + odon. Niassa can be interpreted in two ways: from the Chiyao it means lake [64], and it is also the north-western province in Mozambique. From the Greek, odontos, meaning tooth.

### Diagnosis

As for the type and only known species.

*Niassodon mfumukasi* n. gen. n. sp.

urn:lsid:zoobank.org:act:C27BE4A8-EA64-4132-BABF-B867CC80F397

### Etymology

Mfumukasi, meaning queen in Nyanja. It represents a tribute to the members of the local Nyanja matriarchal society and to all Mozambican women.

### Holotype

ML1620 is a complete skull (Movie S1), mandible, series of 19 dorsal, sacral and caudal vertebrae, ribs, two ilia and a partial femur (Fig. 2,3; Figure S1).

### Type Locality and Horizon

The fossiliferous unit is located near Tulo, a small village situated along the Metangula-Cóbue road (Fig. 1). Tulo is in the Lago District of Niassa Province, northern Mozambique (Fig. 1A). The fossiliferous layer (Fig. 1B, C) is equivalent to L10 from Borges et al.'s [65] “Lunho series” and equivalent to the K5 division of Verniers et al. [30]. The fossil bed is composed of a grey mudstone with abundant septaria-like calcareous concretions [30].

### Diagnosis

A small emydopoid dicynodont (preserved skull length 58 mm; preserved skull greatest width 50 mm); distinguished by the following autapomorphies: radiating pattern of vascular foramina, grooves and ridges on dorsal surface of the frontals, and a weak longitudinal ridge on the dorsal surface of the preparietal. Can be differentiated from Emydops by the larger postfrontals, the presence of a thickened rim around the pineal foramen, the absence of contact between the maxilla and squamosal, the more oval mandibular fenestra which approaches the dorsal and ventral edges of the mandibular ramus, the more rounded profile of the lateral edge of the lateral dentary shelf, the relatively shorter posterior iliac process, the six maxillary teeth and the 11 dentary teeth. Can be differentiated from Myosaurus by the more extensive and strongly developed ornamentation of dorsal surface of the frontals, presence of postfrontals, presence of a thickened rim around the pineal foramen, relatively larger lateral dentary shelf, six maxillary teeth and 11 dentary teeth. Can be differentiated from Compsodon by its smaller size, the absence of a fossa on the facial surface of the maxilla, the absence of contact between the maxilla and squamosal, the dorsal surface of the postfrontals being flush with the dorsal surface of the frontals, the much broader exposure of the parietals on the dorsal surface of the temporal bar, the more vertical orientation of the postorbitals along the lateral edge of the temporal bar, the squared off profile of the junction between the zygomatic and quadrate processes of the squamosal in posterior view, and the six maxillary teeth. Can be differentiated from Dicynodontoides by smaller size, the presence of postfrontals, the absence of a longitudinal ridge along the midfrontal suture, the much broader exposure of the parietals in the temporal bar, the more vertical orientation of the postorbitals along the lateral edge of the temporal bar, the large contribution of the preparietal to the rim of the pineal foramen, the absence of a dentary lamina that obscures the mandibular fenestra in lateral view; acetabulum located ventral to the iliac blade as opposed to posterior to the iliac blade, the six maxillary teeth and the 11 dentary teeth. Can be differentiated from all cistecephalids by the more laterally-directed orbits, the more robust postorbital bar, the more rounded zygomatic arches in dorsal view, squamosals that extend posteriorly past the level of the occipital plate, the relatively more elongate dentary, the larger, more oval mandibular fenestra, the six maxillary teeth and the 11 dentary teeth. Can be differentiated from Cryptocynodon simus by smaller size, the presence of premaxillary teeth, the presence of a more strongly developed caniniform process, the presence of paired depression on the palatal surface of the palatine pad, and a relatively longer interpterygoid vacuity. Can be differentiated from Brachyprosopus by smaller size, the presence of premaxillary teeth, the presence of paired depressions on the palatal surface of the palatine pad, the absence of raised laterally-flaring margins of the interpterygoid vacuity, the absence of a pineal boss, and the more posterior placement of the pineal foramen.

### Remarks

The majority of taxa to which comparisons are made in the differential diagnosis are well-characterized, represented by well preserved specimens, and not subject to outstanding taxonomic problems. *Cryptocynodon simus* Seeley, 1894 is an exception: the only specimen that can be definitely attributed to this species is the fragmentary, poorly preserved holotype (NHMUK PV R2582). Perhaps not surprisingly, taxonomic conclusions about *C. simus* have varied. Various authors [66]–[68] considered the taxon to likely represent *Endothiodon*, whereas others [69], [70] considered the species a junior synonym of *Pristerodon mackayi.* In contrast, we agree with Broom [71] that the holotype most likely represents a distinct, valid species. It can be differentiated from *Endothiodon* by the absence of premaxillary teeth and the absence of depressions on the palatal surface of the palatine. Likewise, it can be differentiated from *Pristerodon* by the more elongate, triangular shape of the palatine pads and the dorsoventrally lower, mediolaterally broader snout.

Interestingly, many of the characteristics of the holotype can be found in a small number of unpublished specimens housed in museum collections in South Africa. SAM-PK-K4534 is a fragmentary skull that preserves almost exactly the same portion of the snout as the holotype, although the quality of preservation and preparation of the former specimen is much better. Like the holotype, SAM-PK-K4534 possesses well developed maxillary toothrows that do not extend onto the premaxilla, a broad, dorsoventrally low snout, and palatine pads with the same elongate triangular shape. Two much more complete skulls that also show this combination of features are CGP/1/2215 and CGP S140. If our identification of the latter two specimens is correct, the overall appearance of *C. simus* is quite similar to *Niassodon*, including a broad temporal bar with widely exposed parietals, and a pineal foramen that is located near the posterior margin of the skull roof. Nevertheless, all of the specimens we refer to *C. simus* differ from *Niassodon* in lacking premaxillary teeth and paired depressions on the palatal surface of the palatine pad, and in having a larger body size (e.g., CGP/1/2215 has a basal skull length of approximately 96 mm). The three referred specimens also indicate that the caniniform process was very weakly developed in *C. simus*, in contrast to the more prominent caniniform process present in *Niassodon*, and the two CGP specimens demonstrate that the interpterygoid vacuity was relatively shorter in *C. simus*. A full description of *C. simus* is beyond the scope of this paper but should be undertaken in the future because the species might have important implications for our understanding of emydopoid phylogeny.

## Anatomical Description

### Nasals

The paired nasals are broken anteriorly at the level of the nasal boss (Fig. 3C,E,F, 5A,B,C). A clear, interdigitated midline suture between the nasals is visible and extends the entire length of the elements (Fig. 5A,B). The remains of the nasal boss suggest that it is a single median swelling with a continuous posterior margin, comparable to the morphology observed in taxa such as *Diictodon* (Fig. 5C, D). The surface of the nasal boss is somewhat rugose and bears small vascular foramina, presumably associated with a keratinous covering [26]. The nasals meet the frontals along a transverse but somewhat interdigitated nasofrontal suture, which is located posterior to the margin of the nasal boss (Fig. 5B, D). Laterally, the nasals contact the prefrontals and lacrimals on the skull roof, and they contact the maxillae on the lateral surface of the skull (Fig. 5C).

### Prefrontals

The prefrontals are small, crescentic elements that make up part of the anterior portion of the skull roof and orbital rim (Fig. 5A, B, C). Anteriorly, they contact the nasals near the posterolateral margin of the nasal boss (Fig. 5B, C). The prefrontals meet the lacrimals on the facial surface of the skull and in the interior of the orbit by means of a straight contact (Fig. 5B, C). Further posteriorly, the prefrontals have a small contact with the frontals along the margin of the orbit (Fig. 5C). There is no prefrontal boss. As in other emydopoids, the prefrontals contribute to the well-developed anterior orbital margin, which extends medially towards the midline of the skull.

### Frontals

The frontals are subtrapezoidal and form a significant portion of the skull roof (Fig. 5A, B). They are separated by a fairly straight, but serrated midline suture (Fig. 5B). The frontals have extensive lateral contacts with the prefrontals and postfrontals (Fig. 5C). Posteriorly, the frontals meet the parietals along an oblique suture; their suture with the preparietal is U-shaped and planar (Fig. 5A, B). Because the postfrontals are relatively large, the frontals make a moderate contribution to the orbital rim (Fig. 5C). Posterolaterally, the frontals have a small contact with the postorbital (Fig. 5A, B). Almost the entire ventral surface of the frontals contacts the ethmoid. Posteriorly, the ventral surface of the frontal also bears a flange that contacts the anterior portion of the descending flange of the parietal (Fig. 5A). The most distinctive feature of the frontals is a radiating pattern of vascular foramina, grooves, and ridges on their dorsal surfaces (Fig. 5B, D). The center of this ornamentation is located near the mid-length of the frontal and is slightly offset laterally, with the ridges and grooves radiating anteriorly and posteriorly. On each frontal the two most strongly developed vascular grooves extend anteriorly and are nearly coincident with the prefrontal-frontal suture. This ornamentation on the frontals is relatively unique among dicynodonts. A similar morphology is present in one specimen of *Myosaurus gracilis* (BP/1/4269), although most specimens of *M. gracilis* seem to lack it.

### Postfrontals

The right postfrontal is completely preserved but only the medial part of the left one remains (Fig. 5A, B). The dorsal surface of the right postfrontal has undergone extensive cracking, but most of its original features can be determined. It is relatively large and triangular, and tapers posteriorly (Fig. 5B). The postfrontal makes a large contribution to the orbital rim (Fig. 5C), and has extensive, relatively planar contacts with the postorbital and frontal. The surface of the postfrontal is flush with the surrounding skull roof, and is not elevated above the frontals as in *Compsodon helmoedi* [72].

### Postorbitals

The right postorbital is much more completely preserved than the left, although its dorsal surface is somewhat cracked anteriorly (Fig. 5A, B). The middle section of the right postorbital bar is missing (Fig. 5D), but the remaining portions of the bar demonstrate that it was relatively thin, unornamented, and not anteroposteriorly expanded (Fig. 5B). The ventral end of the postorbital widens anteroposteriorly, and rests upon a well-developed dorsal spur of the jugal, which separates the postorbital bar from the zygomatic process of the squamosal (Fig. 3E). More posteriorly, the postorbital extends along the entire length of the temporal bar (in contrast to the condition in *Kombuisia* [73] where it has extensive, gently curving contacts with the postfrontal and parietal, and forms the lateral edge of the temporal bar (Fig. 5B, C). The temporal portion of the postorbital faces laterally and dorsally, giving a biplanar configuration (Fig. 5B). The descending laminae of the parietals on the ventral surface of the skull, together with the ventral surface of the postorbitals, form an elongate adductor fossa (Fig. 5A). The contact between the postorbital and parietal/frontal is slot-like (*sensu* [74]). The posterior end of the postorbital contacts the squamosal ventrally, and the tabular medially (Fig. 3B).

### Preparietal

The preparietal is a subrectangular, median element (Fig. 5A, B). It is most easily distinguished in the µCT scans, and our description is based primarily on those data. Anteriorly it contacts the frontals. It is limited by the parietals posteriorly, and it contacts the ethmoid ventrally (Fig. 5A, B). Weak ridges extend along its lateral edges on the dorsal surface of the skull, and an additional weak longitudinal ridge is present near its midline (Fig. 5B). The preparietal forms the anterior half of the margins of the pineal foramen (Fig. 5A, B). The pineal foramen itself is located far posteriorly on the skull roof and is slightly oval in shape with a weakly thickened rim (Fig. 5B).

### Parietals

The dorsal surface of the posterior parietals has undergone extensive cracking (Fig. 5B). Anteriorly the parietals contact the frontals along a serrate suture and also make a point contact with the postfrontal (Fig. 5A, B). The parietals are bounded by the postparietal and tabular posteriorly (Fig. 3B). Anterormedially, the parietals contact the lateral edges of the preparietal, and form the posterior half of the pineal foramen (Fig. 5B). There is no mid-parietal suture posterior to the pineal foramen, perhaps on account of the very posterior location of the pineal foramen (Fig. 5B). As in *Myosaurus*, the parietals have a laterally broad but anteroposteriorly short exposure on the skull roof, such that they only form the posterior portion of the dorsal surface of the temporal bar. Anteriorly, the dorsal surface of the parietals is flat and flush with the surrounding bones of the skull roof, but posteriorly the midline region of the parietals near the pineal foramen is somewhat depressed (Fig. 5B, D). This gives the posterolateral portions of the parietals and postborbitals a somewhat ridge-like appearance. The descending flange of parietal, forming the medial wall of ventral adductor fossa, is vertically-oriented, thickened at the base, but blade-like for most of its ventral extent (Fig. 5C). The epipterygoid abuts on the base of the posterolateral surface of the descending flange of the parietal. The parietal also has a ventral contact with the anterior part of the supraoccipital.

### Lacrimal

The lacrimal is more completely preserved on the left side of ML 1620 (Fig. 5A, B). It has a small facial exposure that contacts the nasals anteriorly, the maxilla ventrally and the prefrontal dorsally (Fig. 5B, C). The lacrimal forms most of the internal anterior margin of the orbit, and bears a large, oval lacrimal foramen located at the anteroventral corner of the orbit (Fig. 5B, C). Dorsal to the lacrimal foramen there is a second small foramen that opens anteriorly into the interior of the nasal cavity. This foramen does not connect with the naso-lacrimal duct. The lacrimal forms an anteroposteriorly short portion of the floor of the orbit, and contacts the jugal posteriorly (Fig. 3E).

### Premaxilla

The anterior end of the specimen is damaged, such that the tip of the snout is missing and the nasal cavity is exposed in transverse section (Fig. 2, 3). The nasal cavity is sub-rectangular in shape. None of the facial portions of the premaxilla are preserved, although part of its contribution to the secondary palate is visible (Fig. 6C). The µCT data reveal several aspects of the morphology of the palatal portion of the premaxilla. The premaxilla contacts the maxilla laterally along a sinusoidal suture (Fig. 6C). More medially, it contacts the anterior tip of the palatines, and also forms the anterior border of the oval choanae (Fig. 6C, D). A median palatal ridge extends to the vomer, and articulates with the latter element by way of a V-shaped recess (Fig. 6C). On the dorsal surface of the premaxilla there is a triangular ascending process that articulates loosely with the vomer posteriorly (Fig. 6D, E).

Three tooth positions are present in the premaxilla, the more posterior of which is located at the sinusoidal suture with the maxilla (Fig. 6C, E). In both premaxillae only the posterolateral tooth is erupted; the other teeth are present but unerupted (Fig. 6C). Given the location of the third tooth position, the third premaxillary tooth is partially surrounded by the maxilla. An unerupted tooth bud is in contact with medial surface of the root of the erupted tooth in each premaxilla. The premaxillary and maxillary teeth are similar in shape and size but differ from the mandibular teeth, but both differ from the dentary teeth in being cylindrical and tapering to a pointed tip. The pulp cavity extends about three-quarters of the way through the tooth. Wear facets appear to be present on the anteriormost maxillary teeth on both sides of the skull.

### Maxillae

The anterior ends of both maxillae have been lost because of the damage to the front of the skull (Fig. 3E, F, 6A, B). The left maxilla is more completely preserved, and a smoothly curved rim along its preserved anterior margin represents the posterior edge of the external narial opening (Fig. 6A, B). As preserved, the left maxilla has a triradiate shape, with a dorsal process that contacts the nasal and lacrimal; a posterior process that extends towards the zygomatic arch and contacts the jugal; and the ventrally-directed caniniform process (Fig. 6A). The caniniform process is gracile and located at about the same level as the anterior orbital margin (Fig. 6A). A postcaniniform keel arises from the posterior surface of the caniniform process and extends to the posterior edge of the maxilla (Fig. 6C). Caniniform tusks are not erupted and the maxillary antrum is a rounded cavity that projects into the suborbital bar. The facial surface of the maxilla bears several well-developed vascular foramina that are likely associated with the presence of a keratinous beak [26].

The palatal portion of the maxilla is visible using the µCT data (Fig. 6C). The maxilla contacts the premaxilla anteriorly along a sinusoidal suture. It also contacts the palatine medially and the ectopterygoid and jugal posteriorly. The maxilla bears a well-developed row of six teeth that are oriented anteromedially to posterolaterally, parallel to the maxilla-palatine suture, and that is continuous with the premaxillary tooth row. Three replacement teeth also are visible in each maxilla in the µCT data, and these teeth would have erupted medial to the previous generation of teeth. The erupted and replacement teeth are associated in groups of three: two erupted teeth laterally and one unerupted tooth medially. The three groups of teeth in each maxilla are clearly separated by interalveolar septa. There is a small tooth bud near the anterior end of the left maxillary antrum, which may represent a developing tusk. Alternatively, it may represent a pathological tusk, such as the unusally-shaped unerupted tusk reported in a specimen of the large cryptodont *Odontocyclops* (AMNH FARB 5566) [75]. Posterior to the maxillary antrum there is a small foramen that pierces the maxilla posterodorsally, resembling the condition in *Emydops* [76], although in ML 1620 this is only visible on the left maxilla. However, a labial fossa bounded by the jugal, maxilla, and palatine is absent.

### Jugal

The jugal is not completely exposed on either side of the specimen, but much of its morphology can be determined from the µCT scan (Fig. 3E). It forms a significant portion of the floor of the orbit and suborbital bar. Anteriorly, it contacts the lacrimal, and it is overlapped laterally by the maxilla (Fig. 3E). The right side of the specimen demonstrates that the jugal separates the maxilla from the squamosal. On this side of the specimen, the posterior tip of the posterior process of the maxilla is somewhat damaged, but a triangular fossa is present on the lateral surface of the jugal that represents the scarf joint between the jugal and maxilla (Fig. 3E). The squamosal slots into a similar triangular fossa on the posterior third of the suborbital bar, but it does not reach the maxillary fossa on the jugal. The jugal also bears a tapering dorsal process that extends a short distance up the posterior side of the postorbital bar medially (Fig. 3E).

### Squamosal

As is typical for dicynodonts, the squamosal primarily consists of three processes, a dorsal process that contributes to the occipital plate and the posterior margin of the temporal fenestra, a zygomatic process that extends anteriorly, and a quadrate process that extends ventrally and supports the quadrate-quadratojugal complex (Fig. 7A, C). The dorsal process slopes dorsomedially, and contacts the postorbital on the lateral surface of the skull (Fig. 7C). On the occipital plate, the dorsal process contacts the tabular and supraoccipital, and forms the lateral third of the margin of the postemporal fenestra (Fig. 7E). The zygomatic process of the squamosal arises from near the midpoint of the element's height, and is dorsoventrally broadened but thin lateromedially (Fig. 7A, B, C, D). The junction between the zygomatic and quadrate processes of the squamosal has a relatively squared-off profile in posterior view (Fig. 7E), similar to most emydopoids but differing from the notched profile seen in most other dicynodonts. The zygomatic process tapers to a pointed tip anteriorly, and contacts the jugal along a scarf joint (Fig. 3E, 7A). However, the better preserved right side of the specimen suggests that there is no contact between the zygomatic process of the squamosal and postorbital or the squamosal and the maxilla. The quadrate process of the squamosal extends ventrally (Fig. 7E), and its lateral surface bears the fossa for the origination of M. adductor mandibulae externus lateralis, e.g. [77]. On the left side, a sinuous suture between the squamosal and the quadratojugal lies in the muscular fossa, and the quadrate rests in a fossa on the anterior surface of the quadrate process (Fig. 7B). Medially, the quadrate process has a long vertical and horizontal contact with the surface of the paroccipital process (Fig. 7E, F).

### Quadratojugal

The quadratojugal is a subtriangular, plate-like element that is concave anteriorly (Fig. 7B, F). The dorsal end inserts into the squamosal. The ventral end is much narrower and abuts the dorsal surface of the lateral condyle of the quadrate. The quadratojugal forms the lateral wall of the quadrate foramen.

### Quadrate

The quadrate possesses typical dicynodont morphology with two articular condyles separated by a midline groove (Fig. 7D), and a rounded dorsal process that rests in a fossa on the squamosal (Fig. 7F). The lateral articular condyle is relatively thick dorsoventrally and is D-shaped in ventral view (Fig. 7D), with a convex lateral edge and a straight medial edge. The lateral condyle is anteroposteriorly shorter than the medial articular condyle (Fig. 7D). The medial condyle is narrower than the lateral condyle, elongate, and curves slightly laterally towards its posterior end. The medial and lateral condyles are separated by a relatively wide, well-demarcated groove that is of consistent width along its anteroposterior extent (Fig. 7D). The medial surface of the medial articular condyle has a flat crescent-shaped surface for the articulation of the lateral end of the stapes (Fig. 7F). The articular surfaces of the condyles are at about the same height, in contrast to emydopoids like *Dicynodontoides* or *Myosaurus*, in which they are slightly offset. This gives the articular surface a somewhat shoe-shaped profile in posterior view (Fig. 7E), comparable to that seen in some specimens of *Emydops* (e.g., SAM-PK-11060) but differing from the more L-shaped profile in *Myosaurus* (e.g., SAM-PK-3526). In the dorsal process of the quadrate there is a posteromedial excavation for the articulation of the quadrate ramus of the pterygoid. Posterior to this excavation, separated by a shallow ridge, the opisthotic contacts on the majority of the medial part of the quadrate (Fig. 7F). The quadrate is only co-ossified with the quadratojugal, which likely allowed some mobility of the element.

### Stapes

Only the left stapes, measuring 7.9 mm in length, is preserved and it is disarticulated from the skull. Medially, the stapes is roughly conical, narrowing to a minimum diameter of 1.2 mm cross-section at the midpoint of its length (Fig. 7A). As the stapes tapers laterally it flattens dorsoventrally to form a spatulate articular facet with the quadrate (Fig. 7E, F). The lateral facet is crescentic, with a concave dorsal surface and a convex lateral surface (Fig. 7D). The medial facet is subcircular (Fig. 7A) measuring 4.6 mm in its longer axis and 4.4 mm in its shorter axis. The obliquely-oriented lateral facet measures 4.9 mm in its maximum extension and is 1.0 mm thick. The stapes does not possess a stapedial foramen or any dorsal process.

### Postparietal (Interparietal)

Although the term “interparietal” is common in dicynodont literature we opted to use term “postparietal” to designate the bone as homologous to the mammal postparietal, *sensu* [78]. The suture between the parietals and the postparietal is located at the posteriormost edge of the temporal bar, although this region is somewhat fragmented (Fig. 7C, E). As such, the postparietal does not contribute to the skull roof. However, the postparietal (and the rest of the occipital plate) does slope anteriorly near its dorsal margin (Fig. 7A, B), in contrast to the more vertical arrangement described for *Emydops oweni* [79]. The postparietal-supraoccipital suture forms an inverted U-shaped curve medially (Fig. 7C). It is likely that the postparietal contacts the tabular laterally, but this area of the skull is heavily damaged (Fig. 7E).

### Tabular

The tabular is a plate-like element (Fig. 7C, E). Damage to the specimen has nearly destroyed the left tabular, and the right is poorly preserved. However, as preserved the tabular appears to be relatively large and oval to fusiform in shape (Fig. 7E). Dorsally, the tabular contacts the parietal and postorbital, and medially it contacts the postparietal, supraoccipital and squamosal (Fig. 7E).

### Opisthotic

The opisthotic is a stout bone that contributes to the occipital plate and forms the ventral third of the posttemporal fenestra (Fig. 7E). Laterally, it hosts the squamosal and quadrate in a concavity on its lateral projection, the paroccipital process (Fig. 7D). Anteriorly the opisthotic contacts two bones: the prootic dorsomedially (Fig. 7D) and the basisphenoid ventromedially (Fig. 3C). Posteriorly, it contacts five bones: the supraoccipital dorsomedially, the exoccipital medially, the basioccipital ventromedially, the quadrate ventrolaterally and the squamosal laterally (Fig. 7E). Medially, the opisthotic completely surrounds the wall of the horizontal semicircular canal. The opisthotic forms the dorsal third of the fenestra ovalis.

### Basioccipital, Exoccipital

The occipital condyle is trilobate (Fig. 7E), consisting of fused portions of the basioccipital and the exoccipitals. Note that we segmented it as part of the basioccipital in the µCT rendering images because sutures between the elements were not visible. The dorsal lobes are formed by the exoccipital and the ventral lobe by the basioccipital (Fig. 7E). The articular facet of the occipital condyle is convex but excavated medially by a central occipital pit (Fig. 7E). The exoccipitals are also fused to the opisthotic portion of the occiput (Fig. 7E). At its base, the occipital condyle is flanked by the anteromedially-oriented jugular foramina. The basioccipital tubera have the shape of a right triangle in posterior view (Fig. 7E). The vertical fenestra ovalis on the lateral surface of the tuber forms the upright of the triangle, whereas the medial wall slopes towards the midline of the skull at an angle of 52° (Fig. 7E). The fenestra ovalis faces laterally and is bordered by a thickened rim of bone, the anterior half of which is formed by the basisphenoid (Fig. 3C). The left fenestra has a diameter of 5.3 mm while the right is 5.1 mm in diameter. Between the two basioccipital tubera there is no intertuberal ridge, instead there is a rounded excavation.

A pair of foramina are visible at the base of the exoccipital, corresponding to the XII cranial nerves. The anterior foramen is anteromedially-orientated while the posterior foramen is dorsomedially-oriented.

### Palatines

As is typical of dicynodonts, the palatine can be divided into two sections, a ventral palatine pad that contributes to the secondary palate, and a plate-like dorsal section that forms part of the roof of the choana and surrounds the nasal passage (Fig. 6C). The palatine pad is anteroposteriorly elongated, subtriangular in shape, and tapers anteriorly (Fig. 6C). This morphology, along with the presence of two rounded depressions on the palatal surface of the pad (Fig. 6C), gives the palatines an appearance somewhat like those of *Endothiodon* [80]. The ventral surface of the pad is ornamented by a finely textured surface of pits and small striae. The palatine pad contacts the premaxilla medial to the anterior teeth. It also contacts the maxilla anterolaterally, and the ectopterygoid posterolaterally (Fig. 6C). The plate-like portion of the palatine contacts the jugal and the pterygoid. The palatines are separated medially by the vomer. There are two foramina in each palatine (Fig. 6C). Anterior to the palatine depressions there is one subcircular palatal foramen that is oriented posterolaterally and that communicates with the ventral opening of the internal nares. This foramen is continued as a groove on the medial side of the dorsal flange of the palatine and ends at the level of the maxilla-jugal-ectopterygoid juncture. Finally, at the lateral edge of the anterior tip of the palatine there is another foramen that is oriented dorsally.

### Ectopterygoid

The ectopterygoid is a short element that can be seen in ventral and lateral views (Fig. 6 and 6). Anteriorly, it contacts the maxilla and jugal, whereas posteriorly it contacts the palatine and pterygoid, although it does not extend further posteriorly than the palatines. Near the midpoint of its length, the ectopterygoid forms the lateral wall of a small lateral palatal foramen (Fig. 6,8). The lateral palatal foramen is located near the posterior end of the palatine pad, but it is at about the same level as the pad. This differs from taxa such as *Pristerodon* or *Endothiodon*, in which the foramen is located dorsal to the level of the pad.

### Epipterygoid

The epipterygoid consists of two portions, the ascending ramus and the footplate (Fig. 6A, B). The ascending ramus is a thin rod of bone that extends anterodorsally to contact the descending flanges of the palatine. The footplate is anteroposteriorly expanded and its posterior end is bluntly rounded. The anterior end may have tapered to an anterior point, but it is not well preserved on either side of the specimen (Fig. 6,8).

### Pterygoid

The pterygoid shows the typical dicynodont morphology, with an anterior palatal ramus, a median plate and a posteriorly projecting quadrate ramus (Fig. 6,8). The palatal ramus contacts the palatines and vomer medially, and anteriorly it interdigitates with the ectopterygoid (Fig. 6A, B, C, 8A, B, C, D). The posterior portion of the ventral edge of the palatal ramus bears a low keel that converges with the crista oesophagea on the median plate (Fig. 8D). As in most dicynodonts, there is no lateral pterygoid flange. The angle between the palatal ramus and the sagittal plane of the skull is about 19°, measured at the median plate. The quadrate ramus is considerably thinner than the palatal ramus. The quadrate ramus angles 48° laterally relative to the sagittal plane of the skull at the posteriormost edge of the interpterygoid vacuity. The sub-trapezoidal median plate of the pterygoid is formed by ankylosis of both pterygoids, and it solidly contacts the parasphenoid posteriorly. Both the palatal and the quadrate rami are straight. The interpterygoid vacuity is teardrop-shaped, widening posteriorly (Fig. 6C, 8D).

### Vomer

The vomers are fused and the vomerine septum is relatively wide and bears a shallow trough (Fig. 6C). The posterior median palatal ridge of the premaxilla continues into the ventral crest on the vomerine septum (Fig. 6E). This crest bifurcates posteriorly at the level of the anterior border of the palatines, forming the lateral edges of the median trough. The medial walls of the nasal passages are formed by the vomer, and it forms the roof of the choana posteriorly (Fig. 6C). The vomer has a point contact with the medial wall of the anterior ramus of the pterygoid and forms the anterior half of the margin of the interpterygoid vacuity (Fig. 6A, B, C, 8A, B).

### Anterior Plate (Ethmoid)

The anterior plate is a sheet-like median bone that broadens dorsally into an anteroposteriorly-oriented vacuity (Fig. 8). The plate is considered to be composed of the fused mesethmoid and orbitosphenoid ([81]; see [22] for a review of the interpretations of these elements in dicynodonts). The anterior plate contacts the vomer and palatines ventrally, the postfrontals laterally and the frontals and preparietal dorsally. The dorsal vacuity housed the olfactory bulbs (Fig. 8E).

### Parasphenoid

The parasphenoid is composed of the plate-like, triangular parasphenoid rostrum anteriorly, and by a stout median plate posteroventrally (Fig. 6, 8). The parasphenoid rostrum is anteriorly expanded and tapers posteriorly. The median plate of the parasphenoid is co-ossified with the pterygoid anteriorly and the basisphenoid and the prootic posteriorly (Fig. 8C). The ventral surface of the parasphenoid is perforated by the paired internal carotid canals (0.9 mm in diameter) (Fig. 6D).

### Basisphenoid

The basisphenoid forms a large portion of the basicranium (Fig. 3C). It is composed of two lateral buttresses that contact the opisthotic, the basal tubera of the basioccipital posteriorly and probably the exoccipitals. The lateral buttresses are stout and have slightly convex lateral borders. The prootic rests on the dorsal surface of the basisphenoid (Fig. 8A, C). The basisphenoid forms the anterior border of the fenestra ovalis and surrounds most of the cochlea. There is a dorsomedially-oriented foramen piercing the lateral side of the basisphenoid near the confluence of the opisthotic and prootic. In ventral view, the intertuberal groove separates the lateral buttresses (Fig. 8A, D).

### Supraoccipital

The supraoccipital is a large bone forming the majority of the dorsal portion of the occipital plate (Fig. 7C, E). It is saddle-shaped and has two subtriangular lateral alae with a dorsal median lobe (Fig. 7C). The dorsal and lateral borders of the oval-shaped foramen magnum are formed by the supraoccipital, as are the dorsal borders of the circular posttemporal fenestrae, which are positioned at mid-height of the foramen magnum (Fig. 7E). About midway between the dorsal border of the foramen magnum and the distal edge of the supraoccipital, the element is pierced by two pairs of small foramina. On the left side to ventrolaterally-oriented grooves lead away from the foramina, whereas on the right side dorsally-oriented grooves lead away from the foramina (Fig. 7E). The anterior portion of the supraoccipital surrounds the posterior semicircular canal and the crus commune.

### Prootic

The main body of the prootic is D-shaped in anterior view, and with a short anterior process that supports the pila antotica (Fig. 8A). The pila is long, anterodorsally-directed and rod-shaped with an expanded base. The main body of the prootic is co-ossified with the supraoccipital posteriorly. Laterally, the prootic bears a plate like crest. Behind this crest, a canal extends from the supraoccipital to the level of the prootic (Fig. 8C).

### Sclerotic ossicles

A total of 13 sclerotic ossicles are preserved, although they have been displaced such that they lie below the orbit, close to the pterygoids (Fig. 8 A, B, C, D, E). The ossicles are thin, sub-rectangular sheets of bone, and although some are still in articulation, they no longer form a complete scleral ring.

### Dentary

The mandible is disarticulated from the skull, being slightly displaced posteriorly and laterally such that its anterior margin is in contact with the caniniform process of the left maxilla (Fig. 3A, B, E, F). The dentary is well preserved and is a single element; no suture between the dentaries is visible (Fig. 9C). From the symphyseal region to the mandibular fenestra there are a number of well developed vascular foramina, likely associated with the presence of a keratinous beak (Fig. 9A, B). Anteriorly, the tip of the dentary is rounded and, in dorsal view, the symphyseal region presents a central oval groove bordered laterally by the two dentary tables (Fig. 9F). There is a well developed posterior dentary sulcus (*sensu* [82]) that extends posterior to the tooth row (Fig. 9F). Ventrally, the anterior portion of the dentary contacts the splenial, and the posterior portion rests in a shallow fossa on the lateral surface of the angular (Fig. 9E, G, H). Posteriorly, the lateral surface of the dentary bifurcates into two processes to form the anterodorsal and anteroventral margins of the mandibular fenestra, with the dorsal process being longer and more robust (Fig. 9A, B, G, H). On this process, a strong lateral dentary shelf overhangs the anterior portion of the mandibular fenestra (Fig. 9B, F). Its lateral edge has a smoothly rounded contour in dorsal view, with the apex of the curvature being posteriorly located. This morphology contrasts with the more triangular shape of the shelf in *Emydops*, but is somewhat similar to that of *Myosaurus*. The lateral dentary shelf is relatively thick dorsoventrally, and bears a shallow depression on its dorsal surface. The mandibular fenestra is large and is bounded anteriorly by the dentary, dorsally by the dentary and surangular, ventrally by the angular, prearticular and dentary, and posteriorly by the angular and surangular (Fig. 9A, B, G, H). It is located approximately midway along the length and height of the mandibular ramus, and is oval in shape.

The dentary teeth are organized in a row near the medial margin of the dentary, and replacement teeth appear to erupt medial to the previous generation of teeth (Fig. 9F, 10). The roots are cylindrical with a circular transverse section that gradually changes apically to form a chisel-like crown that is laterally compressed (Fig. 10). However, the resolution of the CT scans is not high enough to determine whether the teeth were serrated. The pulp cavity is conical in shape, widest at its base, and extends approximately three quarters of the total height of the tooth (Fig. 10). Surprisingly, the teeth are open-rooted.

### Splenial

The splenials are fused into a single spur-shaped element that is mostly exposed on the posterior surface of the symphysis (Fig. 9D). It is composed of two posterior subtriangular rami and a median body (Fig. 9D). Anteriorly, it bears a short, lanceolate process that contributes to the ventral portion of the mandibular symphysis. Each ramus bears a vertical crest that extends along the entire length of its dorsal border. The splenial contacts the angular posteriorly along a planar suture (Fig. 9G, H). Dorsal to the symphyseal portion of the splenial, and unattached to the mandible, there is a triangular bone with a rod-like anterior tip, possibly a hyoid fragment (Fig. 9D). A small shaft-like bone was identified as a hyoid in *Daptocephalus* [83].

### Surangular

The surangular is a kinked crescentic bone with a ventral keel on its posterior half (Fig. 9H). It forms the posterodorsal portion of the mandibular fenestra. It also contacts the dentary anteriorly, the angular lateroventrally, and the prearticular and articular posteriorly. At a lateral crest extends along the length of the surangular at the junction between its lateral and dorsal surfaces (Fig. 9H). The anterior end of the bone tapers into a conical process that fits tightly into a deeply excavated sulcus on the dentary. The dorsal surface of the posterior half of the surangular is flat and tilted laterally. The sheet-like ventral keel has an anterior projection and, is completely enclosed by the angular laterally and the prearticular medially (Fig. 9H).

### Angular

The angular is a plate-like bone that forms the ventral margin of the mandibular fenestra, as well as the ventral border of the postdentary portion of the mandible (Fig. 9A, B, G, H). Anteriorly, it bears a shallow fossa in which the ventral posterior process of the dentary rests. Dorsally, the angular contacts the surangular, and it extends posteriorly to contact the prearticular-articular complex (Fig. 9G, H). The reflected lamina of the angular is preserved only on the left side of the mandible, and there it is somewhat fragmentary (Fig. 9B, H). It arises at the level of the posterior margin of the mandibular fenestra, is quite thin, and appears to have an unornamented lateral surface.

### Prearticular-Articular complex

The prearticular and articular are firmly co-ossified (Fig. 9A, B, E, F, H). The articular appears to have the morphology typical of dicynodonts, with two elongate articular surfaces separated by a median ridge (Fig. 9B, D, E, F). A short, blocky retroarticular process is located posterior and ventral to the articular surfaces (Fig. 9D). The articular contacts the surangular and angular anteriorly (Fig. 9G, H). The prearticular is a subrectangular plate-like bone, and is poorly preserved. It contacts the dorsal flange of the splenial anteriorly, the angular and surangular laterally (Fig. 9G, H).

### Atlas

The two atlas neural arches and the atlas intercentrum are preserved (Fig. 12; Movie S1). They are disarticulated from each other and from the skull and remaining vertebral column (Fig. 11). In lateral view, the atlas neural arch is composed of a crested lateral process, a bulbous proatlas facet and a crescentic transverse process (Fig. 12 H, I, J, K). On the anterior portion there is the flat facet for the odontoid laterally and the concave, rounded facet for the exoccipital medially. The left atlas neural arch is completely preserved, but the right one is missing a portion of the transverse process (Fig. 12 L, M, N). The atlas intercentrum is a saddle-like element with two lateral alae for the atlas neural arch facets (Fig. 12 L, M, N).

### Vertebrae

In addition to the atlas-axis complex, a total of 18 vertebrae are partially or fully preserved (Fig. 11, 12). Seventeen of these constitute an articulated section of the vertebral column, consisting of 10 presacral vertebrae, four sacrals, and three anterior caudals (Fig. 11). Only the anterior portion of caudal 2 is preserved. The remaining vertebra is disarticulated from the rest of the column. This vertebra appears to be an anterior caudal based on the preserved portions of the vertebral column (Fig. 11 and 12A–G).

As preserved, the articulated dorsal vertebrae are exposed in ventral view. The centra are somewhat more anteroposteriorly elongate (Fig. 11C) than is often the case for dicynodonts (e.g., compare with the vertebrae of *Eosimops* [82]), implying a relatively flexible vertebral column. The vertebrae are strongly amphicoelous, such that the articular facets of a given centrum have a conical appearance with only a thin septum of bone separating them within the centrum (Fig. 12 G). The dorsal surface of the centrum is remarkably excavated, forming a tetrapyramidal pit (Fig. 12E). In lateral view, there is a pleurocoel formed by two oval perforations aligned anteroposteriorly. The perforations are interconnected medially and ventromedialy-oriented. The pleurocoel extends dorsally to the pedicles (visible in a coronal section). The pedicles are firmly co-ossified with the centra (Fig. 12B, C, D). At mid-length of the centrum, the neural canal is piriform in cross-section but subcircular near the articular facets (Fig. 12B, D). The rectangular neural spines are slightly tilted posteriorly (Fig. 11B, 12C). Near the base of the neural spine there is a slight anteroposterior constriction (Fig. 11B). In a coronal section, the apex of the neural spine is expanded giving a bulbous appearance. The most anteriorly preserved vertebrae have horizontally-oriented zygapophyses (Fig. 12B). The fourth-to-last and third-to-last presacral vertebrae are 0.80 mm wide and 0.76 mm long, whereas the corresponding vertebrae in *Eosimops newtoni* (BP/1/6674) are1.29 mm and 1.11 mm, respectively. The centra are waisted and the articular facets have prominent rims of well-finished bone laterally (Fig. 11C, 12B, D).

There are four sacral vertebrae (Fig. 11A). Like the dorsal vertebrae, the centra are waisted and have well developed rims on the articular facets, but they are anteroposteriorly shorter (Fig. 11C). The sacrals decrease rapidly in length, with the fourth sacral being about 60% of the length of the first. The articulation for the first sacral rib occupies about half the length of the first sacral centrum, but it occupies nearly the entire length of the centrum in the remaining three sacrals (Fig. 11C).

The two preserved caudal vertebrae are slightly smaller than the fourth sacral. The second caudal is poorly preserved. Part of the neural arch and anterior zygapophysis is visible in lateral view. The length of the neural arch is about 75% of the length of the centrum.

### Ribs

Twelve dorsal ribs are preserved in articulation with the vertebral column (Fig. 3B). The distal portions of the articulated ribs are missing, but the proximal portions are sinusoidal in cross section. The dorsal ribs are posteriorly-directed, but this might be slightly exaggerated by preservation of the trunk in a flexed position. The capitulum is an elongated, rod-shaped ventral projection, and the tuberculum is a stout, short process marking the angle with the distal portion of the ribs.

The four sacral ribs are waisted and expand laterally to contact the ilium (Fig. 11B, C). They are shorter and more robust than the dorsal ribs, as is typical for dicynodonts. The first pair of sacral ribs is slightly posteriorly-directed, but the remaining three pairs are transversely-oriented (Fig. 11A, C).

### Pelvic Girdle

All the elements of the pelvic girdle are co-ossified (Fig. 13). Both ilia are preserved, but the left is more exposed in lateral view. The anterior iliac process is relatively short, and the anterior margin of the ilium is gently curved and nearly vertically-oriented (Fig. 13A). This morphology is similar to that observed in *Eosimops* [82], but differs from the more strongly curved anterior margin present in taxa with strongly developed anterior processes (e.g., *Rhinodicynodon* [84]). The posterior iliac process is anteroposteriorly short, but tall dorsoventrally (Fig. 13A). The dorsal edge of the ilium is convex and unnotched. The lateral surface of the iliac blade is gently concave, and bears numerous radial striations. The acetabular portion of the ilium is narrower than the blade, and is located ventral to it (Fig. 13B), in contrast to the more posterior location of the acetabulum in *Dicynodontoides* (e.g. [85]). The left ilium possesses a flared rim from the acetabulum and an acetabular notch (Fig. 13). The pubis is a triangular bone contributing to the ventral part of the acetabulum, whereas the ischium forms the posterior third of the acetabulum (Fig. 13).

### Femur

The right femur is broken, but the proximal portion of the femur is preserved in articulation with the ipsilateral acetabulum (Fig. 14). The head of the femur is ellipsoidal and does not present any medial expansion or trochanter. The diaphysis is slightly waisted (Fig. 14).

## Bone Internal Morphology

In addition to the µCT scan (see methods), we collected histological data from a SRµCT scan of the femur and pelvic girdle (Fig. 14). The femur preserves the epiphysis and the proximal portion of the diaphyseal shaft. It is mostly composed of trabecular bone that is enveloped by a thin periosteal cortex. Indeed, the cortex is remarkably thin compared to the condition typically observed in dicynodonts (e.g., [56]). Forming a concave line, a denser mesh of trabeculae (Fig. 14) separates the epiphyseal region from diaphysis. This gives the area an appearance somewhat similar to the junction between the bony epiphysis and the diaphysis in mammals (e.g., compare to figures in [86]), although in mammals epiphysis and diaphysis are separated by a layer of cartilage instead of denser trabecular bone. Similar to the femur, the bones of the pelvic girdle are composed of trabecular bone and a thin periosteum.

Using the µCT data the pelvic girdle seems co-ossified without conspicuous sutures (Fig. 13). However, the sutural marks between the bones are still visible in the SRµCT images (Fig. 14).

## Neuroanatomy and Inner Ear Morphology

### Brain

We assume that cranial endocast volume provides, with limitations, an approximate and reliable estimate of brain size (see methods section). Therefore, the term “brain” will be used as a synonym of the digitally segmented cranial endocast.

The brain is subtriangular in lateral view and has a volume of 1062 mm^3^. The hindbrain is more expanded than the forebrain (Fig. 15A, C, D). The brain bears a large epiphyseal nerve, and tapers into the olfactory nerve anteriorly (Fig. 15D, E, F). The olfactory bulb is demarcated from the cerebrum by an olfactory tract (Fig. 15E). The olfactory bulb is expanded posteriorly and tapers into the olfactory nerve anteriorly (Fig. 15E). The olfactory tract, bulb and nerve are delimited by the ethmoid (Fig. 15B). The orbits are located far anteriorly relative to the olfactory bulbs (Fig. 15A, B). The cerebrum is subtriangular in lateral view and its dorsal region expands into the epiphyseal nerve (Fig. 15D, E). The cerebrum is delimited by the posterior part of the frontals, preparietal and the anterior portion of the parietals (Fig. 15A, B). The interhemispheral fissure is not visible. The forebrain and the midbrain are separated by a faint cerebral flexure (Fig. 15D). The small optic lobes are nearly indistinguishable from the rest of the midbrain, but compose its anterior portion. The midbrain is formed by a large hypophysis ventrally (Fig. 15E). The midbrain is separated from the hindbrain by a well-marked isthmus (Fig. 15D). The cerebellum is mostly delimited by the supraoccipital and postparietal dorsally, the epipterygoids laterally, and the basisphenoid, parasphenoid and prootics ventrally (Fig. 15A, B). The paraflocculus (10 mm^3^ volume) projects posterolaterally from the lateral walls of the cerebellum (Fig. 15D). The paraflocculi are surrounded by the prootics, supraoccipital and opisthotics, entering through the anterior semicircular canal. The osseous labyrinth is lateral to the cerebellum (Fig. 15D). An unpronounced inflection (pontine flexure) separates the cerebellum and the medulla posteriorly (Fig. 15C).

### Cranial nerves and vascular system

The trigeminal nerve (CN V) arises near the paraflocculi anteroventrally (Fig. 15E). The trigeminal nerve passes through the embayment formed between the pila antotica base and the alar portion of the prootic close to the vena capitis lateralis. The vena capitis lateralis seems to have passed along the prootic lateraloventrally in a horizontal orientation.

Two sulci on the ventral wall of the parietal suggest that in the dorsal surface of the brain endocast two rims converge posteriorly to the epiphyseal nerve. In turtles, the vena capitis dorsalis bifurcates at approximately the same region of the brain [87], so these structures may be homologous in *Niassodon* (Fig. 15D). In *Niassodon*, the path of the vena capitis dorsalis is partially lost because no fossilized structures are preserved between the epiphyseal nerve and the prootic. However, this vein would probably pass along the majority of the dorsal surface of the prootic groove, joining the vena capitis lateralis at its posterior end. The fusion of these two veins would exit the brain through the posttemporal fenestra [22].

The facial nerve (CN VII) is directed laterally and passes between a notch on the suture between the prootic and basisphenoid, forming the facial foramen (Fig. 8A, B). This position for the facial foramen is conserved in other dicynodonts such as *Endothiodon* [88] and *Lystrosaurus* [22]. Although its complete path is not entirely visible, it seems that the facial nerve arises from the ventral portion of the lateral side of the brainstem. The facial nerve (CN VII) exits through the facial foramen on the ventral portion of the alar portion of the prootic (Fig. 15E). The preserved portion of the facial nerve is short and passes at the same level as the horizontal semicircular canal. The vestibulocochlear nerve (CN VIII) exits from the dorsal portion of the cerebellar auditory bulbus, medial to the anterior semicircular canal (Fig. 15E), and divides into the cochlear and vestibular nerves. The cochlear nerve is located lateral to the vestibular nerve. The vestibular nerve pierces the prootic vertically and reaches the anterior ampulla. The cochlear nerve has an irregular course that passes through the prootic, descends through the basisphenoid and eventually reaches the cochlea. The glossopharyngeal and the vagoaccessory nerves (CN IX, X, XI) exit the hindbrain laterally at nearly midheight (Fig. 15E). The glossopharyngeal and vagoaccessory nerves exit the brain through the jugular foramen and are surrounded by the exoccipital, basioccipital and opisthotic. This cranial nerve exit touches the cochlea. The hypoglossal nerve (CN XII) pierces the exoccipital, arising from the hindbrain with a mediolateral orientation (Fig. 15E). Two pairs of the hypoglossal nerve perforate the basioccipital in the posteriormost hindbrain.

### Osseous labyrinth

The osseous labyrinth is composed of a vestibule that connects to the vestibular organ dorsally, and to the cochlea ventrally (Fig. 15). The L-shaped vestibule is composed of a short lateromedially-oriented canal that links to the fenestra ovalis, and then slopes into a stout, dorsoventrally-oriented portion. There is no visible separation between the utriculus and sacculus within the vestibule. The cochlea is a conical ventral projection on the vestibule and it curves slightly anteriorly (Fig. 15H). The vestibular organ is composed of three semicircular canals that are subequal in diameter (Fig. 15G, H): the anterior and posterior semicircular canals are of equal thickness, whereas the horizontal semicircular canal is stouter. The horizontal semicircular canal is subcircular and the vertical semicircular canals are ovoid (Fig. 15H). The crus comunis is slightly waisted (Fig. 15H). The ampulla of the posterior semicircular canal is fused to the ampulla of the horizontal semicircular canal forming a short and bulky ventral projection (Fig. 15I), similar to the condition observed in *Cistecephalus* [14] and *Emydops* [19]. The ampulla of the anterior semicircular canal is globular and well-developed, whereas the anterior ampulla of the horizontal semicircular canal is a small expansion on its medial side (Fig. 15I).

## Discussion

### Phylogenetic position

**Figure 16:**
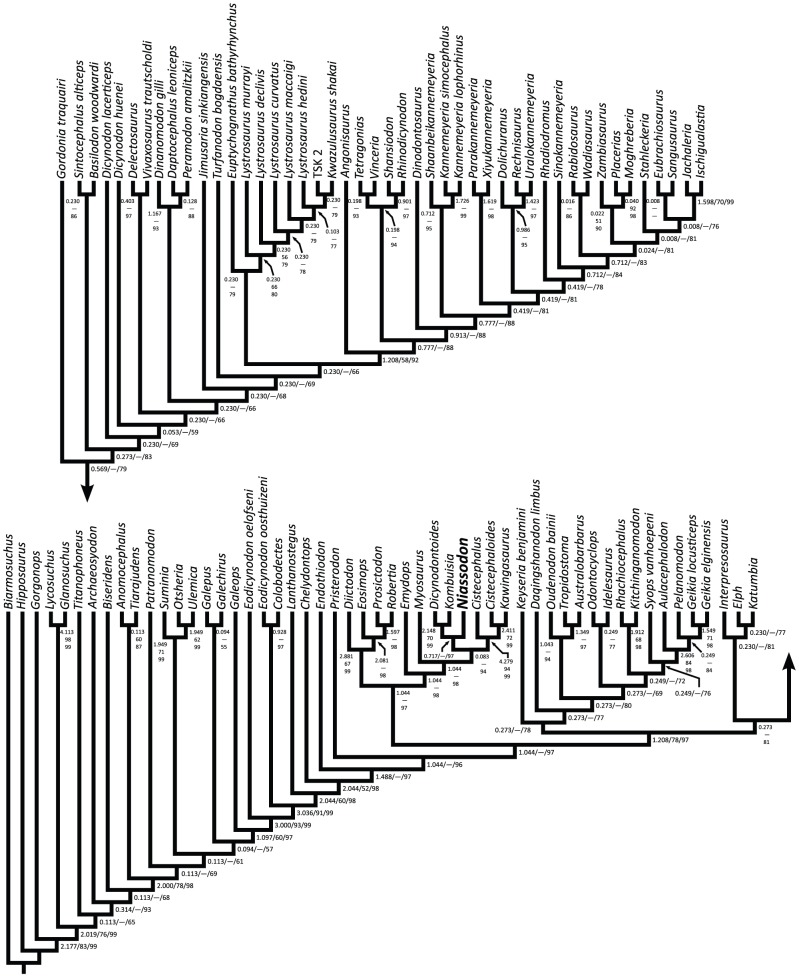
Most parsimonious cladogram from the phylogenetic analysis. Scores: 999.866 steps, consistency index = 0.240, retention index = 0.711. Numbers at nodes represent decay index (left/top), symmetric resampling (middle), and the percentage of the 463,436 suboptimal trees in which the node is resolved (right/bottom). *Niassodon mfumukasi* in bold.

A single most parsimonious tree was discovered by the searches (length 999.866; C.I.  = 0.240, R.I.  = 0.711), and the topological results are shown in Figure 16. Not unexpectedly, the topology of the optimal cladogram is nearly identical to that of Kammerer et al. [48]; the only difference is the more basal position of *Myosaurus* within Emydopoidea. *Niassodon* is reconstructed as the sister taxon of *Dicynodontoides* + *Kombuisia*, making it a member of Kingoriidae (*sensu* [89]). Three discrete-state synapomorphies support this placement: mid-ventral plate of vomers with an expanded, oval-shaped area posterior to junction with premaxilla (character 85, state 0); trough on mid-ventral plate of vomers (i.e., ventral surface concave ventrally with raised edges) present (character 88, state 0); four sacral vertebrae (character 144, state 1). Symmetric resampling and decay support for this placement are relatively low, but this topology is present in 97% of the sample of 463,436 cladograms up to seven steps longer.

The addition of *Niassodon* to the data matrix (Text S1) resulted in a minor rearrangement of the relationships within Emydopoidea. Specifically, *Myosaurus* falls in a more basal position, instead of being the sister group of Kingoriidae [82], [90], [91] or Cistecephalidae [48], [73], [73], [92,93]. This arrangement is somewhat appealing given the superficially similarity of *Emydops* and *Myosaurus.* At the same time, the relatively nested position of *Niassodon* is surprising given its retention of several seemingly basal character states, including the presence of premaxillary teeth, the relatively long maxillary tooth rows, and the long, well-developed posterior dentary sulcus. The eventual inclusion of additional taxa that likely fall within Emydopoidea or just outside of it (e.g., the new Zambian tusked cistecephalid, *Compsodon*, *Cryptocynodon*, *Brachyprosopus*) will help to test whether the position of *Niassodon* in our phylogeny is robust. If *Niassodon* eventually does move to a more basal position within Emydopoidea, it potentially could provide a new, more *Pristerodon*-like model for the ancestral morpholgy for the clade.

### Neuroanatomy and paleobiological implications

Only a handful of dicynodont osseous labyrinths have been described [5], [7], [10], [12], [14], [19], [94] and an unpublished natural endocast is present in the *Aulacephalodon* specimen BP/1/1557. Previous publications described in detail the periotic anatomy of several anomodonts and attempted to understand the morphology of the osseous labyrinth [6], [22]. Comparisons must be made carefully due to the poor preservation of most specimens. On a previous report [14], it was found that the osseous labyrinth of a serially sectioned *Cistecephalus* specimen was deformed, with the horizontal semicircular canal oblique relative to the vertical canals, but the ampullae were well preserved and strongly developed. Similarly, the inner ear in a sectioned *Pristerodon* specimen [12] is anteroposteriorly compressed with the anterior semicircular canal bent ventrally. The osseous labyrinth of *Placerias* examined in [7] is missing the majority of the semicircular canals but the lagena is exceptionally elongate relative to the preserved portions of the semicircular canals. *Placerias* is much larger than *Cistecephalus* or *Pristerodon*, and the fact that the inner ear is relatively small in *Placerias* indicates that the inner ear did not scale isometrically with the skull in dicynodonts. The best preserved inner ears include the *Diictodon* specimen reconstructed in [5] (incorrectly identified as *Dicynodon leoniceps* in that paper), the natural endocast described in [4], [10] (AMNH FARB 6156, potentially referable to *Oudenodon* if Broom's initial identification of the specimen as *Dicynodon bolorhinus* is correct; see [93] for taxonomic details), the reconstruction for *Emydops* by [19], and the natural endocast in BP/1/1557 (*Aulacephalodon*). The general anatomy of the inner ear of the well-preserved specimens is similar to *Niassodon* with some subtle differences. The utriculus is swollen in AMNH FARB 6156 [10], and the crus communis is subtriangular in *Diictodon* [5]. The *Emydops* specimen does not possess a strongly developed ampulla of the anterior semicircular canal, and the semicircular canals seem to have relatively wider diameters [19]. BP/1/1557 possesses a very long lagena, similar to that observed in *Placerias*, again most likely reflecting the much larger size of the specimen compared to *Niassodon*.

The orientation of the horizontal semicircular canals has been commonly used to infer habitual head posture [17], [95], [96]. When the skull of *Niassodon* is oriented such that the horizontal semicircular canal is horizontal, the skull roof slopes slightly anteroventrally, but the snout is not strongly directed towards the substrate (Fig. 15B). This contrasts with the situation in taxa such as *Diictodon*, *Dicynodontoides*, and *Dicynodon lacerticeps* [97], potentially implying that a reevaluation of the head posture in those species might be needed. This orientation of the skull also results in a slight anteroventral angulation of the maxillary tooth row (11.1° relative to horizontal), which has the effect of increasing the occlusion angle between the maxillary and mandibular teeth. Such an arrangement may have been common among smaller, toothed dicynodonts. For example, in another publication the authors oriented the skull of *Pristerodon* in a similar fashion, and the position of the tooth rows in that taxon would result in a similar angulation in this orientation [98]. Because the power stroke in *Pristerodon* (and most other Permian dicynodonts) included both an upwards and posteriorly-directed component of movement, such angulation of the tooth rows may have helped to optimize the amount of time the teeth were in occlusion.

Among therapsids, particular attention has been paid to the brains of therocephalians and cynodonts because of their relevance for the evolution of the mammalian brain [6], [17], [99], [100]. However, a number of authors also have presented data on the brain anatomy of dicynodonts. A problem that affects some of the work on dicynodonts is the fact that many older brain reconstructions in the literature include identifications for specimens based on outdated taxonomy making their relevance to modern dicynodont systematics somewhat obscure. Von Huene [3] figured the posterior section of the brain of *Stahleckeria* based on the internal space in one of the skulls that he described. A partial endocast of *Dicynodon lacerticeps* (identified as *Dicynodon dutoiti*; see [93]) was figured by [2] and he attempted a reconstruction of the brain for comparison with several extant reptiles. Reconstructed brains of three dicynodonts based on serially-sectioned specimens were figured by [6], but he did not provide precise identifications for them. Based on the drawings of the specimens, and their associated locality information, they likely represent *Brachyprosopus* (“Anomodont A”), *Diictodon* (“Anomodont E”), and *Pristerodon* (“Anomodont H”; note that it was tentatively identified this specimen was tentatively identified by [6] as *Emydops,* but the presence of four maxillary postcanines would be usually high for this genus). Others [16], [22] figured an endocranial cast and a brain reconstruction, respectively, of *Lystrosaurus*, and [22] also figured a reconstruction of a brain of an indeterminate species of *Dicynodon sensu lato*. In another publication [11], a brain reconstruction based on a serially-sectioned specimen was ascribed to *Dicynodon*, but based on the drawings of the specimen and its occurrence in the *Tapinocephalus* Assemblage Zone, it likely represents *Diictodon*. The re-identification of these specimens shows that information is available for taxa that span much of dicynodont phylogeny, ranging from basal taxa such as *Pristerodon* to derived Triassic forms such as *Lystrosaurus* and *Stahleckeria.* Despite this broad phylogenetic sampling, dicynodont brain morphology appears to have been relatively conservative within the clade, and comparable in its general organization to the brains of many reptiles. Dicynodont brains are also relatively small for a given body size, again showing more similarities to the situation in reptiles that that in mammals [2], [101]. One important point of variation among dicynodonts brains is the fact that the brain of *Lystrosaurus* appears to have been anteroposteriorly shorter and dorsoventrally deeper than in most other dicynodonts [16], [22], a change that likely reflects the overall shortening and deepening of the skull in that genus. The digital endocast of the brain of *Niassodon* demonstrates that it resembled those of other dicynodonts in retaining an overall conservative morphology. Examples of its “reptile-like” organization include: the presence of a large epiphyseal nerve, the hindbrain being broader than the forebrain, the presence of a large hypophysis, and the narrow, elongate shape in dorsal view. Similar features can been seen in the figured brains of other dicynodonts such as *Lystrosaurus* and *Dictodon* [11], [22], and they are also present in the more derived cynodonts such as *Thrinaxodon* and *Diademodon* [99]. This implies that a reptilian-grade brain morphology was conserved within much of Synapsida from the Permian at least until the late Triassic, confirming the idea that the evolution of an enlarged brain occur late in the evolution of the therapsids [102]. Thus, this gives support to the hypothesis that late expansion of the brain size can be explained by cortical growth and by invasion of the collicular sensory pathways into the isocortex, allowing higher auditory acuity and an improved spatial representation of sound [102].

The preservation of the ethmoid in *Niassodon* validates the tentative reconstruction of the anterior portion of the brain in “*Dicynodon”* [22]. Indeed, the brain morphology of *Niassodon* closely resembles the condition in *Diictodon* or “*Dicynodon”* [11], [22] because of its narrow, elongated shape and the wide angles between the different brain regions, in contrast to the morphology in *Lystrosaurus* [22].

In the endocast of *Niassodon*, the cerebral lobes are not well marked; thus, volumetric measurements could not be achieved. Nevertheless, the total brain (1062 mm^3^) and floccular volumes can be estimated. The degree of correlation between the size of the subarcuate fossa (frequently referred to as the floccular fossa in the dicynodont literature) and the size of the paraflocculus varies in mammals, being relatively weak in marsupials but strong in primates [103]. Assuming a close correlation between the size of the fossa and the size of the paraflocculus, the paraflocculi of *Niassodon* occupy 1.9% of the brain volume. This proportion is relatively large and falls well within the range of extant birds (1–2% [104]). Volumetric data for fossil mammals is rare [105], but another it has been reported that the paraflocculus occupied only 0.2% of the total endocast volume in the mammal *Vincelestes* [106]. A number of authors have discussed the presence or absence of a floccular fossa in dicynodonts (e.g., [107]), with a general trend being noted for the loss of this character in large Triassic dicynodonts. It has been suggested that the presence or absence of this feature is simply a function of body size [17], with small dicynodonts displaying relatively large fossae and large dicynodonts possessing small fossae or lacking them altogether. However, a consideration of the distribution of this character among dicynodonts shows that the relationship may not be so simple. Some large Permian dicynodonts such as *Rhachiocephalus* or *Aulacephalodon* possess floccular fossae [107], whereas some medium-size Triassic dicynodonts that lack a fossa, such as *Tetragonias or Shansiodon*, are comparable in size to Permian taxa that possess one (e.g., *Oudenodon*). If the relatively large paraflocculus of *Niassodon* is not simply a result of scaling relationships, it could imply that the species might have had good visual acuity. An additional hypothesis could be that the this part of the brain was being used to another function or even that it was selected (i.e. enlarged during evolution) due to a developmental constrain indirectly related to the size of the fossa. Further studies are required to disentangle all these possibilities.

### Encephalization Quotient

Our predicted body masses for *Niassodon* ranged from approximately 364 g to approximately 849 g (mean 491 g; see methods above, and Fig. 4) while the endocranial volume has 1062 mm^3^. We assembled relative brain volume *vs.* body mass data from a previously compiled database for mammals and non-mammalian cynodonts [99], and for birds and non-avian dinosaurs [108] (Table S3). *Niassodon mfumukasi* lies on the same regression line as the non-mammalian cynodonts, near the cynodonts *Probainognathus jenseni* and *Thrinaxodon liorhinus* (Fig. 17). Using Eisenberg's encephalization quotient equation [109], *Niassodon* has an encephalization quotient of 0.19, which is comparable to other non-mammalian cynodonts (<0.25, [99]). This result supports the idea that non-mammaliaform synapsid brains did not have an enlarged cortex and cerebellum [99]. This data also shows that in addition to the reptilian-grade morphological traits of *Niassodon*, it also possesses a relative brain volume comparable to non-mammalian cynodonts. Nevertheless, caution should be taken because *Niassodon* is the only non-cynodont synapsid plotted, so the effects of variation at the base of the synapsid tree have yet to be assessed.

**Figure:**
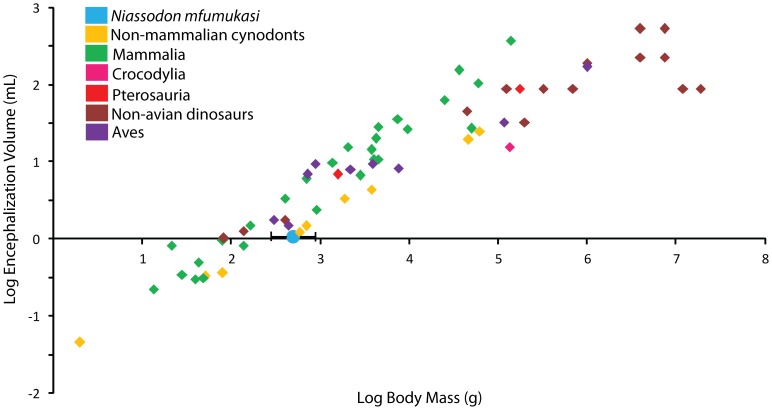
Brain volume to body mass plot in different tetrapod clades.

### Ontogenetic Stage

The holotype of *Niassodon* is a relatively small dicynodont, with an estimated basal skull length of 63 mm. Ideally, the developmental stage of the individual could be inferred by examining its bone microstructure. A true external fundamental system is rare in dicynodonts [110], but several taxa have been reported to show a distinct slowing of growth in presumed adulthood (e.g., [56], [60], [111]–[114]). However, assessment of the bone histology of *Niassodon* is complicated by the fact that only one limb element, the right femur, is present and the diaphysis is not fully preserved. The resolution of the SRµCT data also is not sufficient to resolve histological details in the preserved portion of the cortex of the femur, although new synchrotron-based technologies are being developed that may provide additional information in the future [115]. The SRµCT data do indicate that abundant trabecular bone with a marked orientation is present within the femur. This fact may be indicative of an adult animal [116].

Several other characters provide some additional insight into the ontogentic stage of the holotype of *Niassodon*. First, all bones of the pelvic girdle and several cranial bones (e.g., basioccipital-exoccipital, preparietal-frontal, frontal-prefrontal, parietal-frontal, basioccipital-basisphenoid) are co-ossified or completely fused. The sutures between the pelvic bones can only be distinguished in the SRµCT 3D data, but even using this technique, the degree of fusion in many cranial bones is so high that no evidence of such sutures remains (e.g., basioccipital-exoccipital). Second, the degree of fusion of the neural arches and vertebral centra is commonly used as an ontogenetic indicator in various clades (e.g. [117], [118]), and in *Niassodon* all vertebrae have co-ossified neural arches and centra. Finally, juvenile dicynodont specimens tend to have large orbits relative to the temporal openings (e.g., see figure 1 in [90]). In *Niassodon,* the orbit is about 50% of the length of the temporal opening, a proportion that is more consistent with adult dicynodont skulls than juveniles [90].

Nevertheless, the presence of an unerupted tooth bud enclosed in the left maxillary antrum may suggest that ML1620 could be a juvenile. However, the fact that no tooth bud was found in the right maxilla indicates that this trait could be explained by abnormal growth of an extra tooth or even a vestigial tusk that did not erupt. Dicynodonts can be tusked or tuskless, and most species are very consistent in whether individuals are tusked or tuskless. However, some intraspecific variation related to the tusks has been noted. Other author [119] hypothesized that the presence of tusks was a sexually dimorphic feature in *Diictodon feliceps*, and other authors [75], [113] documented apparently random intraspecific variation in the presence of tusks in *Odontocyclops whaitsi* and *Tropidostoma dubium*, respectively. Additional publications [79], [120] reported on rare occurrences of “double-tusked” dicynodont specimens that possessed two erupted tusks on one side of the skull. The presence of a tusk bud in the antrum of the left maxilla but not in the right in the holotype of *Niassodon* is the first documented instance of such asymmetry in unerupted tusks in a dicynodont. However, given that *Niassodon* is represented by a single specimen, it is difficult to determine whether this observation has any wider paleobiological significance.

Taken together, the available lines of evidence suggest that the type of *Niassodon* represents an adult specimen of a small-bodied dicynodont, not a juvenile of a larger taxon.

### Bone Histological Macrostructure

Although SRµCT data lacked sufficient resolution to distinguish fine details of the bone tissue of the femur, they did uncover a layer of dense trabecular bone located between the diaphysis and epiphysis on the element's proximal end. As noted above, this gives the area of the bone an appearance superficially similar to the junction between the bony epiphysis and diaphysis in mammals, although in mammals this region includes a layer of cartilage instead of denser trabecular bone [86]. The epiphyses of non-mammalian synapsids generally are not thought to have constituted separate centers of ossification, in contrast to the case in mammals. For example, [121] examined the trabecular structure of a polished section of a proximal dicynodont femur, and concluded that the animal likely had a cartilaginous epiphysis on the end of the bone similar to the epiphyses of extant turtles. Other authors [122], [123] stated that they found no gross anatomical evidence of bony epiphyses in their investigations of the postcranial skeletons of the cynodonts *Diademodon* and *Thrinaxodon*, respectively. In our observations of dicynodont postcrania, we have found that juvenile individuals typically have poorly defined joint surfaces on the proximal and distal ends of their long bones, and that these areas usually have a roughened or pitted texture consistent with the presence of a cartilaginous epiphysis. The joint surfaces become better ossified and more defined over the course of ontogeny, but we have not observed obvious gross anatomical evidence of the presence of a separate bony epiphysis (e.g., a suture between an epiphysis and diaphysis; an unfused but clearly ossified epiphysis) in dicynodonts. Despite the extensive recent interest in the bone histology of dicynodonts, almost no studies have examined the histology of the epiphyseal regions of the long bones. Longitudinal sections of the ends of limb bones of *Diictodon* were made by [60] but they provided no description of these sections other than to note that a cartilaginous epiphysis or articular cartilage was not preserved in the elements they examined.

One epiphysis-like structure has been widely reported in dicynodonts. In several species of Triassic kannemeyeriiforms the olecranon process of the ulna initially forms as a separate center of ossification that becomes fused to the rest of the ulna later in ontogeny [3], [124]–[128]. *Placerias* seems to be an exception to this pattern because the olecranon process was apparently unfused throughout life [7], as evidenced by the retention of a separate olecranon process in an extremely large individual of *Placerias* (UCMP A269/25429 and UCMP A269/25432). Most Permian dicynodonts possess very weakly developed olecranons and show no evidence of a separate center of ossification in the area. Even in Permian taxa with large olecranon processes, such as the cistecephalids *Kawingasaurus* [129] and *Cistecephalus* [130], the process seems to be completely contiguous with the diaphysis of the ulna with no suture or other feature to suggest it was a distinct center of ossification. It has been hypothesized by [126] that the separately-ossified olecranon of kannemeyeriiforms represented an independent evolution of bony epiphyses in dicynodonts and suggested they may be present in other elements of their skeletons, although she did not present additional data to support the latter claim. In addition it is known that the olecranon process is not specified in Hox11 mutant mice [131], this increases the interest in adding paleohistological data from basal synapsids to this discussion to test the hypothesis proposed by [132], [133] that traction epiphyses evolved from sesamoid structures.

The discovery of a structure resembling a bony epiphysis in the significantly older and distantly related *Niassodon* suggests that a reevaluation of the epiphyses evolution in dicyndonts may be warranted. It will be especially important to determine whether other taxa show a similar demarcation between the diaphysis and epiphysis, and how its histological structure compares to comparable areas in the limb bones of extant mammals.

### Biostratigraphic correlations between East African Karoo basins

In a previous publication it was proposed that the K5 formation of the Metangula Graben could be correlated with the *Endothiodon* Zone of the South African Karoo Basin [32] based on the occurrence of *Endothiodon* cf. *bathystoma* (also see [33]). What was known as the *Endothiodon* Zone encompasses parts of the *Pristerognathus* and *Tropidostoma* assemblage zones of the current Karoo biostratigraphic subdivisions [134], [135]. However, *Endothiodon* itself does not allow a precise correlation to be made because it is present in the *Pristerognathus*, *Tropidostoma* and *Cistecephalus* zones [136]. *Niassodon* is endemic to the Metangula Graben, so it cannot be used to infer stratigraphic correlations at this time, and other material collected by our team is not yet sufficiently prepared to allow certain identifications.

One specimen that can provide some additional biostratigraphic constraint is BP/1/5749, an isolated but nearly complete humerus that we identify as *Oudenodon*. Although it was collected with cranial and postcranial material referable to *Endothiodon*, several characters of BP/1/5749 indicate that it represents *Oudenodon.* Both *Oudenodon* and *Endothiodon* possess roughly square deltopectoral crests, but that of *Oudenodon* is somewhat longer relative to the overall length of the humerus, and the crest in *Endothiodon* tends to be dorsoventrally thicker. The humeral head is relatively weakly developed in *Endothiodon*, whereas it is more prominent and encroaches on the dorsal surface of the element in *Oudenodon*. The posterior corner of the proximal end of the humerus, where M. subcoracoscapularis inserts, is relatively rounded in *Endothiodon* whereas it is more of a distinct protuberance in *Oudenodon*. Finally, the posterior edge of the mid-shaft region in *Oudenodon* flares out into a well-developed pinna-like process (*sensu* [137]), whereas this process is absent in *Endothiodon*. In all of these features, BP/1/5749 shows a much closer resemblance to known *Oudenodon* specimens than *Endothiodon* specimens from Mozambique or South Africa.

In the Karoo Basin of South Africa, *Oudenodon* first appears in the upper *Tropidostoma* Assemblage Zone and ranges into the *Dicynodon* Assemblage Zone [113]. Its stratigraphic range overlaps with that of *Endothiodon* in the upper *Tropidostoma* zone and the lower portion of the *Cistecephalus* zone, and the co-occurrence of *Endothiodon* and *Oudenodon* in the K5 formation in Mozambique suggest that those rocks were deposited within the time of this overlap (ca. 256 Mya; [138]). This fits well with Verniers et al. 's [30] palynology-based correlation of the K5 formation with the *Tropidostoma* and *Cistecephalus* zones. Based on recent refinements in biostratigraphy [72], [75], [139]–[142] a *Cistecephalus* zone age for the K5 formation also would make it time-equivalent with the Upper Madumabisa Mudstone (Luangwa Basin, Zambia), the Usili Formation (Ruhuhu Basin, Tanzania), and the “Chiweta Beds” (Malawi). Despite the proximity of the Metangula Graben to the Mozambique-Tanzania border, the K5 formation does not continue into Tanzania. However, most of the so-called upper Karoo units in the basin do extend into Tanzania, particularly the KSc and KSe formations, and these units have been tentatively correlated with the Manda beds of the Ruhuhu Basin [30]. Given the surprising diversity of archosaurs present in the Manda beds (e.g., [141], [143]–[145]), it will be important to determine whether the KSc truly is a lateral equivalent of the Manda beds, and if so, whether it preserves a comparable fauna.

## Supporting Information

Figure S1 3D interactive visualization of all preserved bones and internal structures including brain, internal ears, cranial nerves and vasculature of *Niassodon mfumukasi* (ML1620).
(PDF)

Movie S1 Reconstructed skull and atlas of the emydopoid dicynodont *Niassodon mfumukasi* (ML1620) from the Late Permian of northern Mozambique.
(AVI)

Table S1 *Niassodon mfumukasi* (ML1620) skull bone contact matrix (1- contact; 0- do not contact; ?- unknown) and Bone Color Code.
(XLSB)

Table S2 Body mass estimates for dicynodonts based on skull, femur and humerus length.
(XLS)

Table S3 Body mass *versus* encephalization volume data based on Rowe et al. (2011) and Franzosa (2004).
(XLSX)

Text S1 Data matrix coded for *Niassodon mfumukasi* (ML1620) based on Kammerer et al. (2013).
(TXT)
