# Correction: Bringing Dicynodonts Back to Life: Paleobiologyand Anatomy of a New Emydopoid Genus from the Upper Permian of Mozambique

**DOI:** 10.1371/journal.pone.0094720

**Published:** 2014-04-11

**Authors:** The PLOS ONE Staff

There are errors in Figures 6, 7, 11, & 12 and errors in the legends for Figures 2, 5, 6, 7, 8, 9, 12, 13, 14, & 15. The authors have provided corrected figures and legends here.

**Figure 2:**
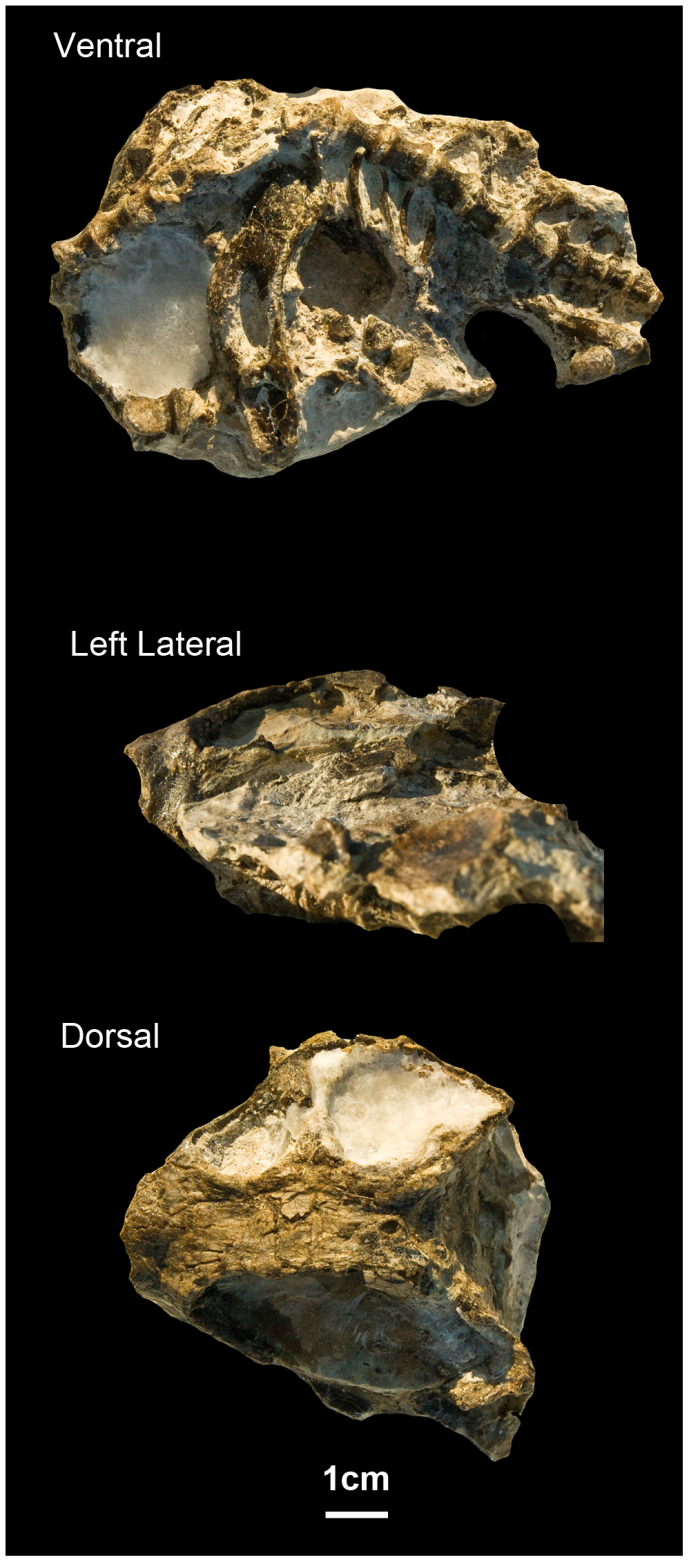
ML1620 (*Niassodon mfumukasi* holotype) in ventral, dorsal and left lateral views. Posterior part of the vertebral column and pelvic girdle were cropped in dorsal view.

**Figure 5:**
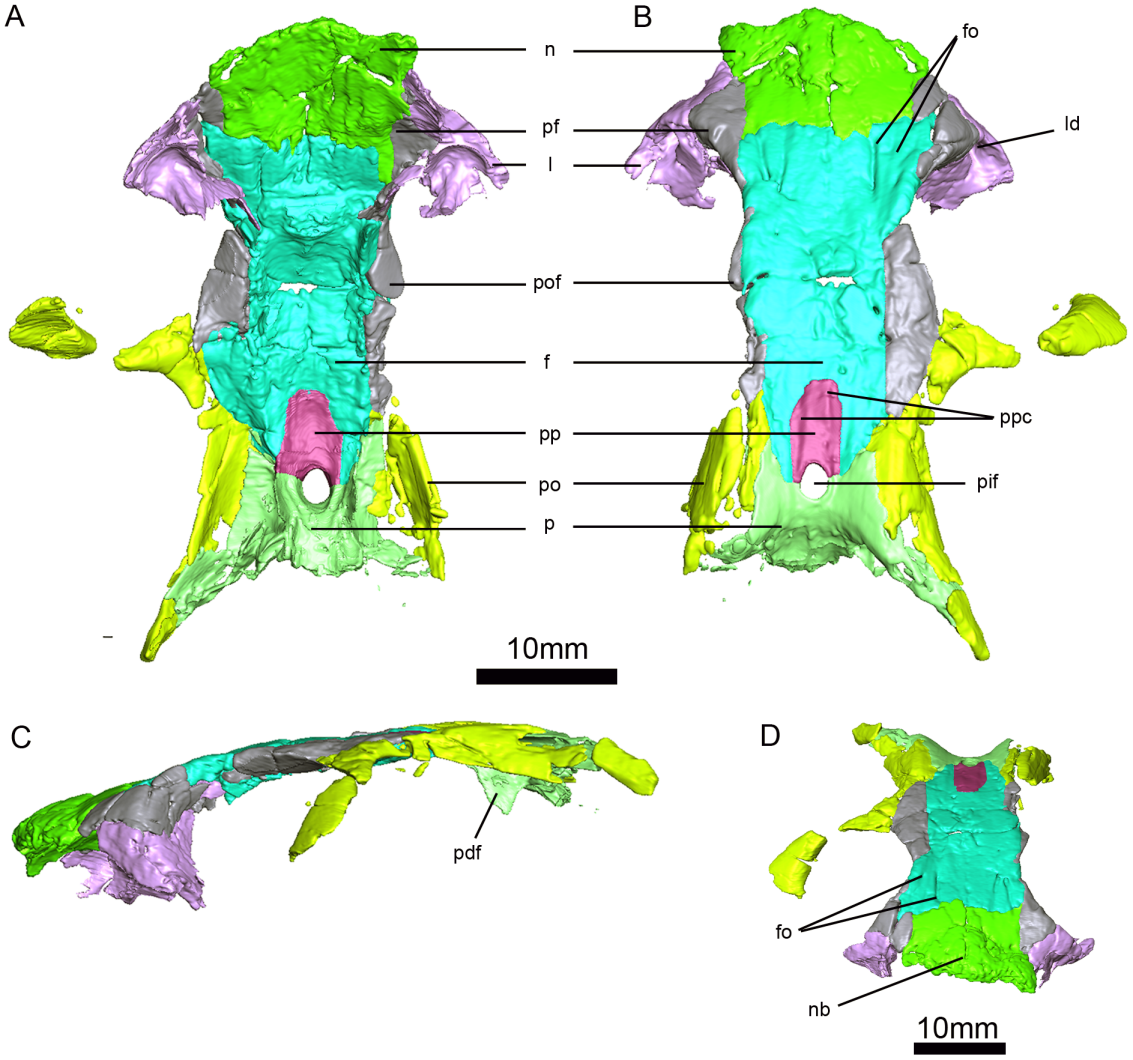
*Niassodon mfumukasi* skull roof. Ventral (A), dorsal (B), left lateral (C), anterodorsal (D) views. **f**, frontal; **fo**, frontal ornamentation (grooves and ridges); **l**, lacrimal; **ld**, lacrimal duct; **n**, nasal; **nb**, nasal boss; **p**, parietal; **pdf**, descending flange of the parietal; **pf**, prefrontal; **pif**, pineal foramen; **po**, postorbital; **pof**, postfrontal; **pp**, preparietal; **ppc**, preparietal crests (forming a weak longitudinal grove between them).

**Figure 6:**
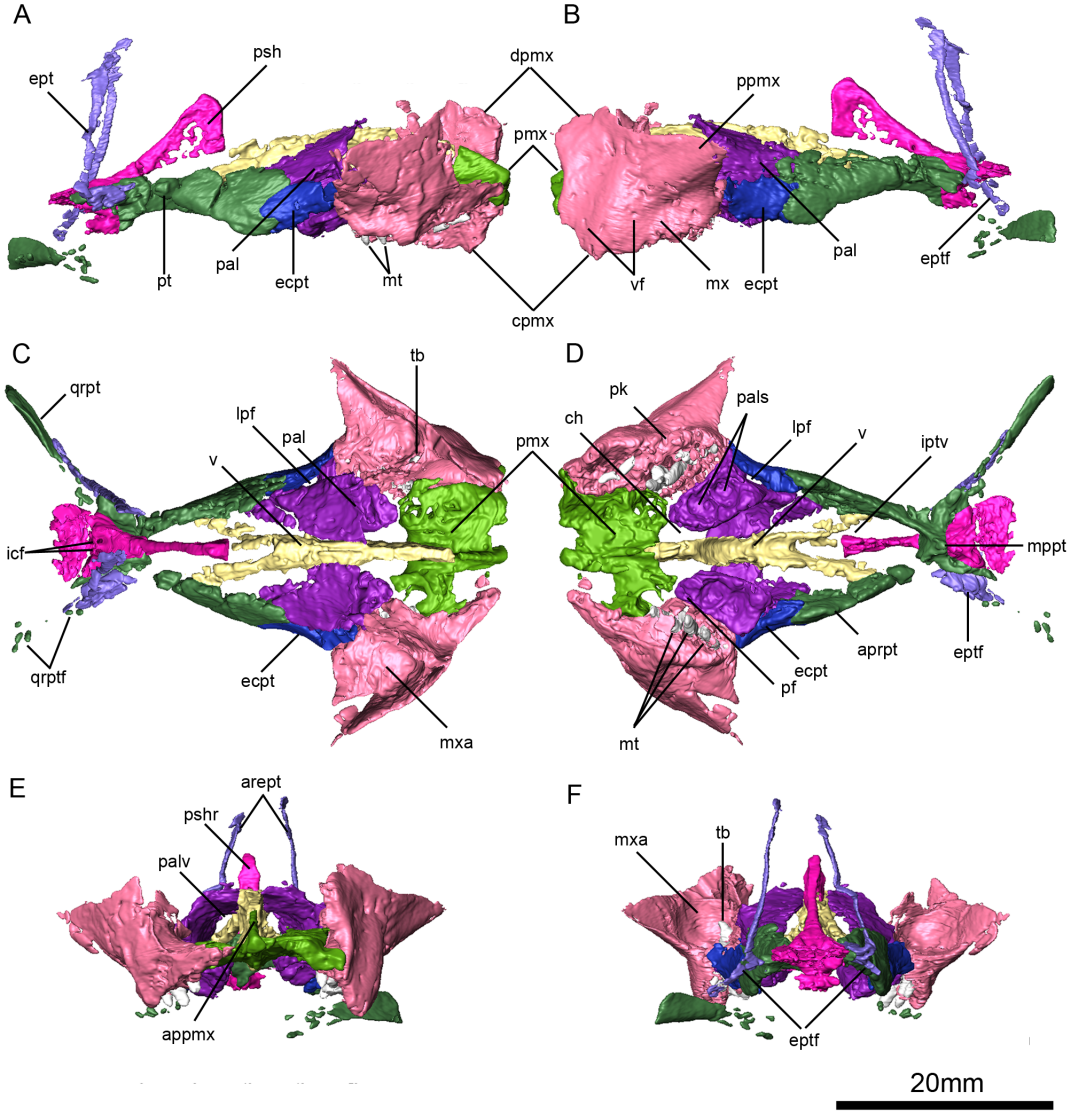
*Niassodon mfumukasi* palate. Right lateral (A), left lateral (B), dorsal (C), ventral (D), anterior (E), posterior (F) views. **appmx**, ascending process of the premaxilla; **aprpt**, anterior palatal ramus of the pterygoid; **arept**, ascending ramus of the epipterygoid; **ch**, choanae; **cpmx**, caniniform process of the maxilla; **dpmx**, dorsal process of the maxilla; **ecpt**, ectopterygoid; **ept**, epipterygoid; **eptf**, epipterygoid foot; **icf**, internal carotid foramina; **iptv**, interpterygoid vacuity; **lpf**, lateral palatal foramen; **mppt**, medium plate of the pterygoid; **mt**, maxillary teeth; **mx**, maxilla; **mxa**, maxillary antrum; **pal**, palatine; **pals**, palatine sulci; **palv**, palatine vacuity; **pf**, palatal foramen; **pk**, postcaniniform keel; **pmx**, premaxilla; **ppmx**, posterior process of the maxilla; **psh**, parashenoid; **pshr**, parashenoid rostrum; **pt**, pterygoid; **qrpt**, quadrate ramus of pterygoid; **qrptf**, quadrate ramus of pterygoid fragments; **tb**, tooth bub; **v**, vomer; **vf**, vascular foramina.

**Figure 7:**
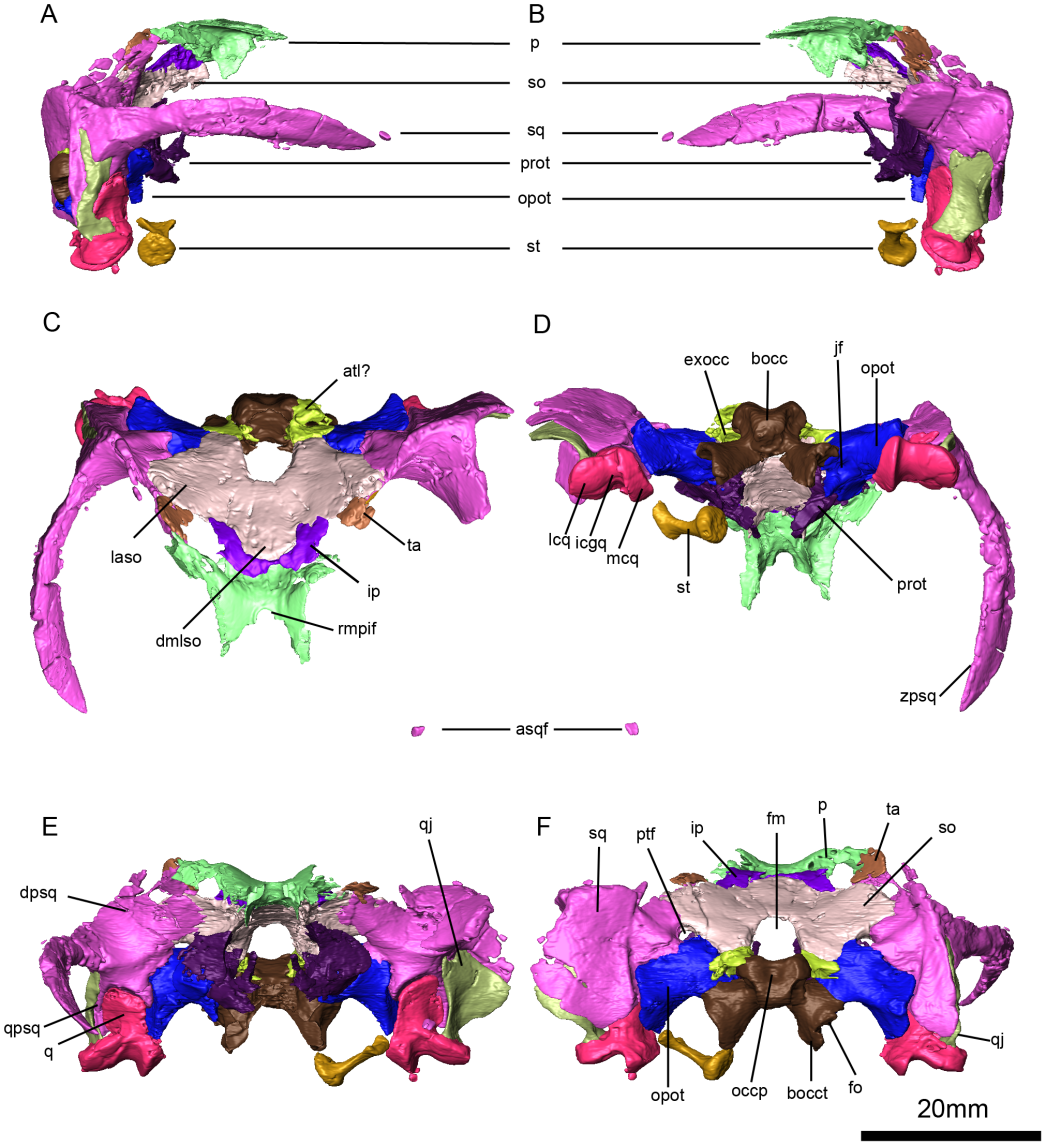
*Niassodon mfumukasi* occipital region. Right lateral (A), left lateral (B), dorsal (C), ventral (D), posterior (E), anterior (F) views. **asqf**, anterior left squamosal fragment; **atl**, parcial atalantal ring; **bocc**, basioccipital; **bocct**, basioccipital tubera; **dmlso**, dorsal median lobe of the supraoccipital; **dpsq**, dorsal process of the squamosal; **exocc**, exoccipital; **fm**, foramen magnum; **fo**, fenestra ovalis; **icgq**, intercondylar groove of the quadrate; **ip**, interparietal (postparietal); **jf**, jugular foramen; **laso**, lateral ala of the supraoccipital; **lqc**, lateral condyle of the quadrate; **mcq**, medial condyle of the quadrate; **occp**, occipital pit; **opot**, opisthotic; **p**, parietal; **prot**, prootic; **ptf**, postemporal fenestra; **q**, quadrate; **qj**, quadratojugal; **qpsq**, quadrate process of the squamosal; **rmpif**, rim of the pineal foramen; **so**, supraoccipital; **sq**, squamosal; **st**, stapes; **ta**, tabular; **zpsq**, zygomatic process of the squamosal.

**Figure 8:**
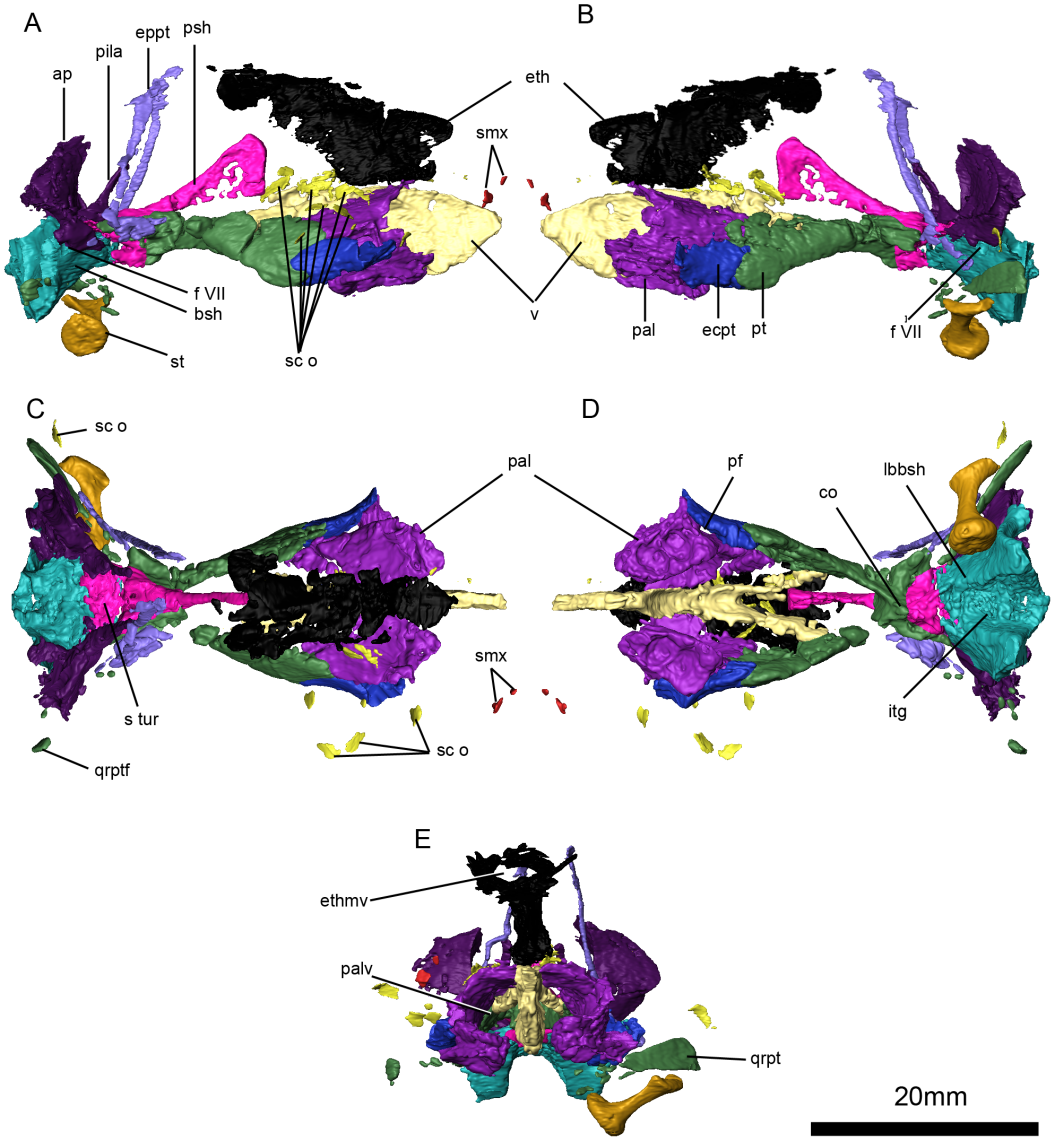
*Niassodon mfumukasi* internal cranial bones. Right lateral (A), left lateral (B), dorsal (C), ventral (D), and anterior (E) view. **ap**, alar process of the prootic, **bsh**, basisphenoid; **co**, crista osophagea; **ecpt**, ectopterygoid; **eppt**, epipterygoid; **eth**, ethmoid; **ethmv**, ethmoid vacuity; **f VII**, facial foramen; **itg**, intertuberal groove; **lbbsh**, lateral buttress of the left basisphenoid; **pal**, palatines; **palv**, palatine vacuity; **pila**, pila antotica of the prootic; **pf**, lateral palatal foramen; **psh**, parasphenoid; **pt**, pterygoid; **qrpt**, quadrate ramus of the pterygoid; **qrptf**, quadrate ramusof the pterygoid fragments; **sc** **o**, sclerotic ossicles; **smx**, septomaxilla; **st**, stapes; **s** **tur**, sella turcica; **v**, vomer.

**Figure 9:**
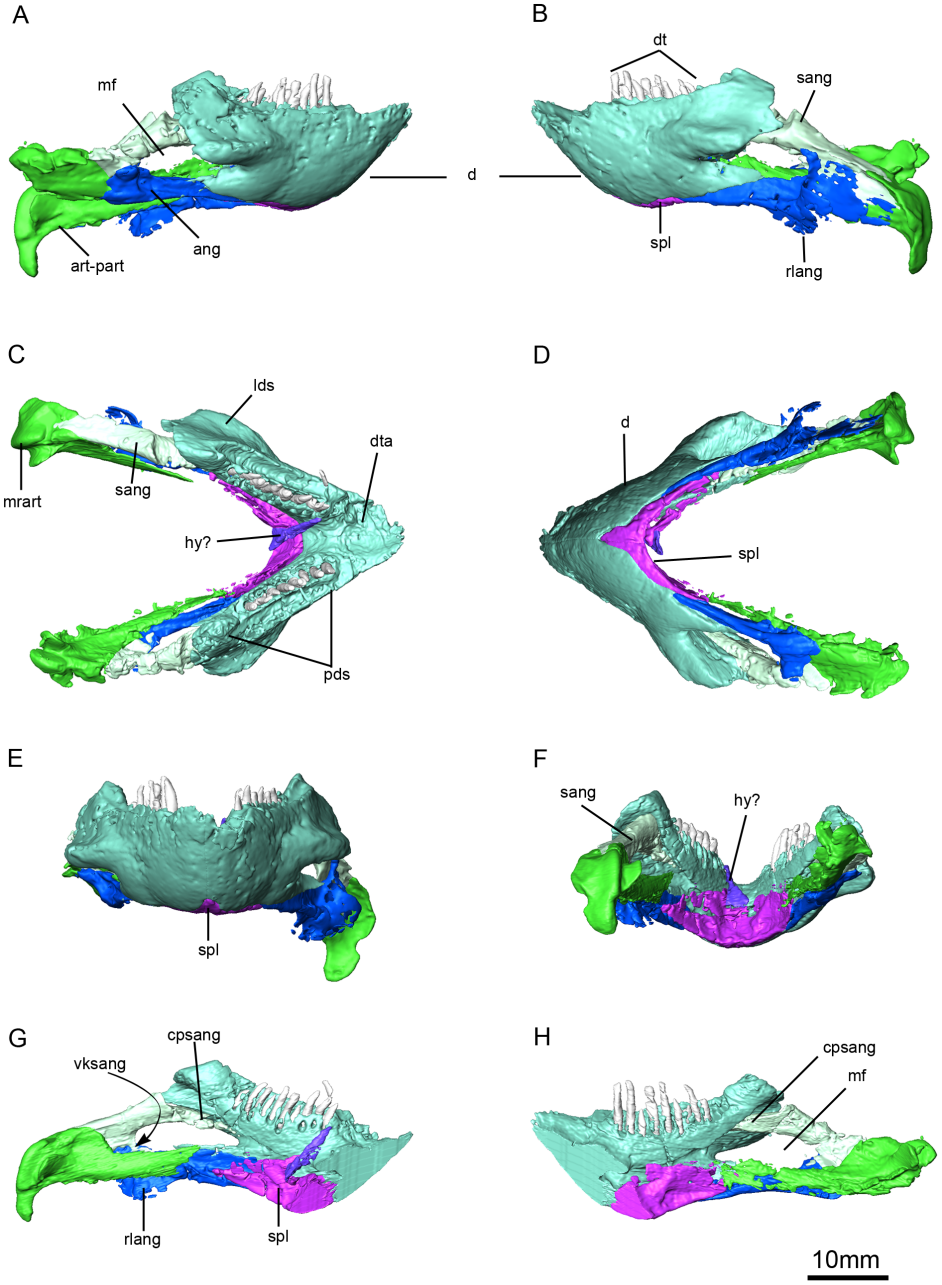
*Niassodon mfumukasi* mandible. Right lateral (A), left lateral (B), dorsal (C), ventral (D), anterior (E), posterior (F) views; left ramus in medial view (G) and right ramus in medial view (H). **ang**, angular; **art-part**, articular-prearticular complex; **cpsang**, conical process of the surangular; **d**, dentary; **dt**, dentary teeth; **dta**, dentary table; **hy?**, probable hyoid; **lds**, lateral dentary shelf; **mf**, mandibular fenestra; **mrart**, median ridge of the articular; **pds**, postdentary sulcus; **rlang**, reflected lamina of the angular; **sang**, surangular; **spl**, splenial; **vksang**, ventral keel of the surangular.

**Figure 11:**
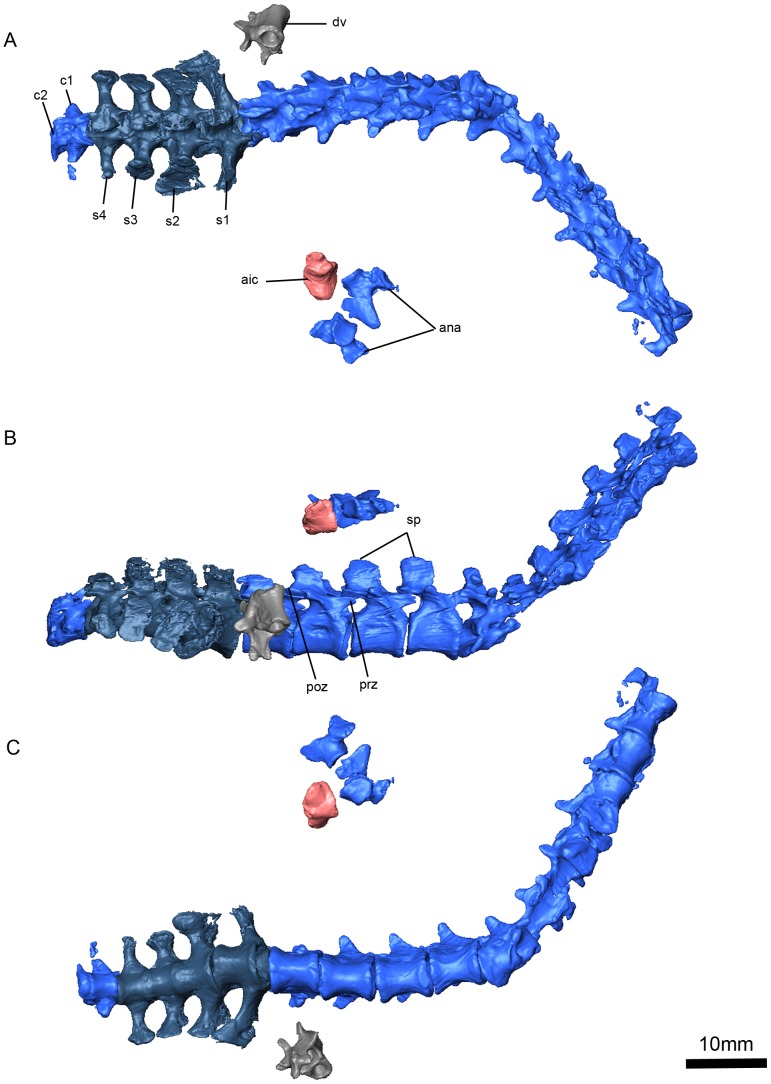
*Niassodon mfumukasi* vertebral column. Dorsal (A), right lateral (B), ventral (C) views. **ana**, atlas neural arches; **aic**, atlas intercentrum; **c**, caudal vertebra; **dv**, dorsal vertebra; **poz**, postzygapophysis; **prz**, prezygapophysis; **s**, sacral vertebra; **sp**, spinous processes.

**Figure 12:**
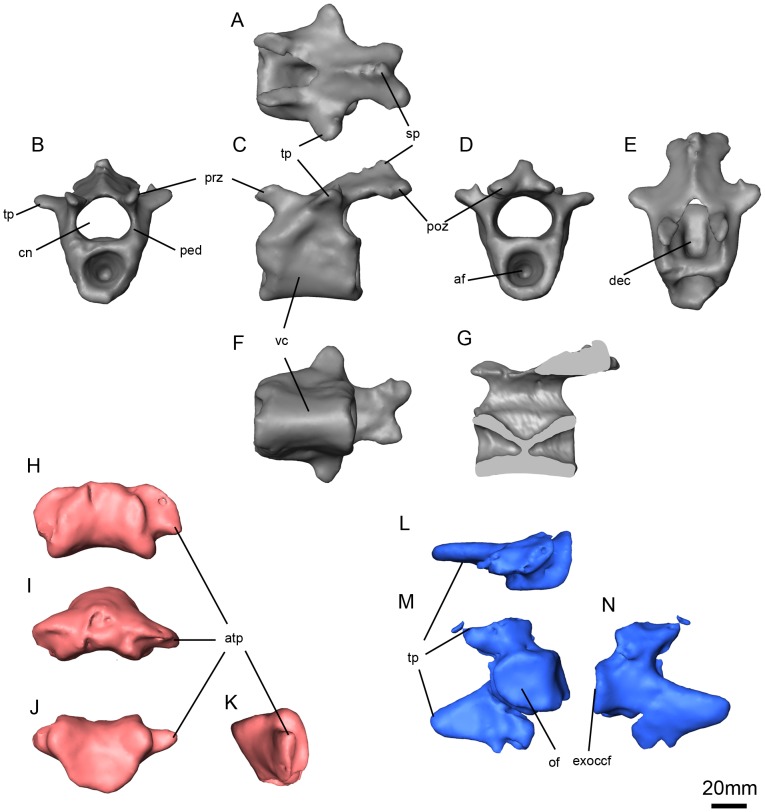
*Niassodon mfumukasi* caudal vertebra and atlas. Caudal vertebra in dorsal (A), anterior (B), left lateral (C), posterior (D), anterodorsal (E), ventral (F) views and sagittal section (G); atlas intercentrum in ventral (H), anterior (I), dorsal (J), right lateral (K) views; left atlas neural arch in dorsal (L), medial (M), left lateral (N) views. **af**, articular facet; **atp**, atlas intercentrum transverse process; **cn**, neural canal; **dec**, dorsal excavation on the centrum; **exoccf**, exoccipital facet; **of**, odontoid facet; **ped**, pedicle; **poz**, postzygapophysis; **prz**, prezygapophysis; **sp**, spinous process; **tp**, transverse process; **vc**, vertebral centrum.

**Figure 13:**
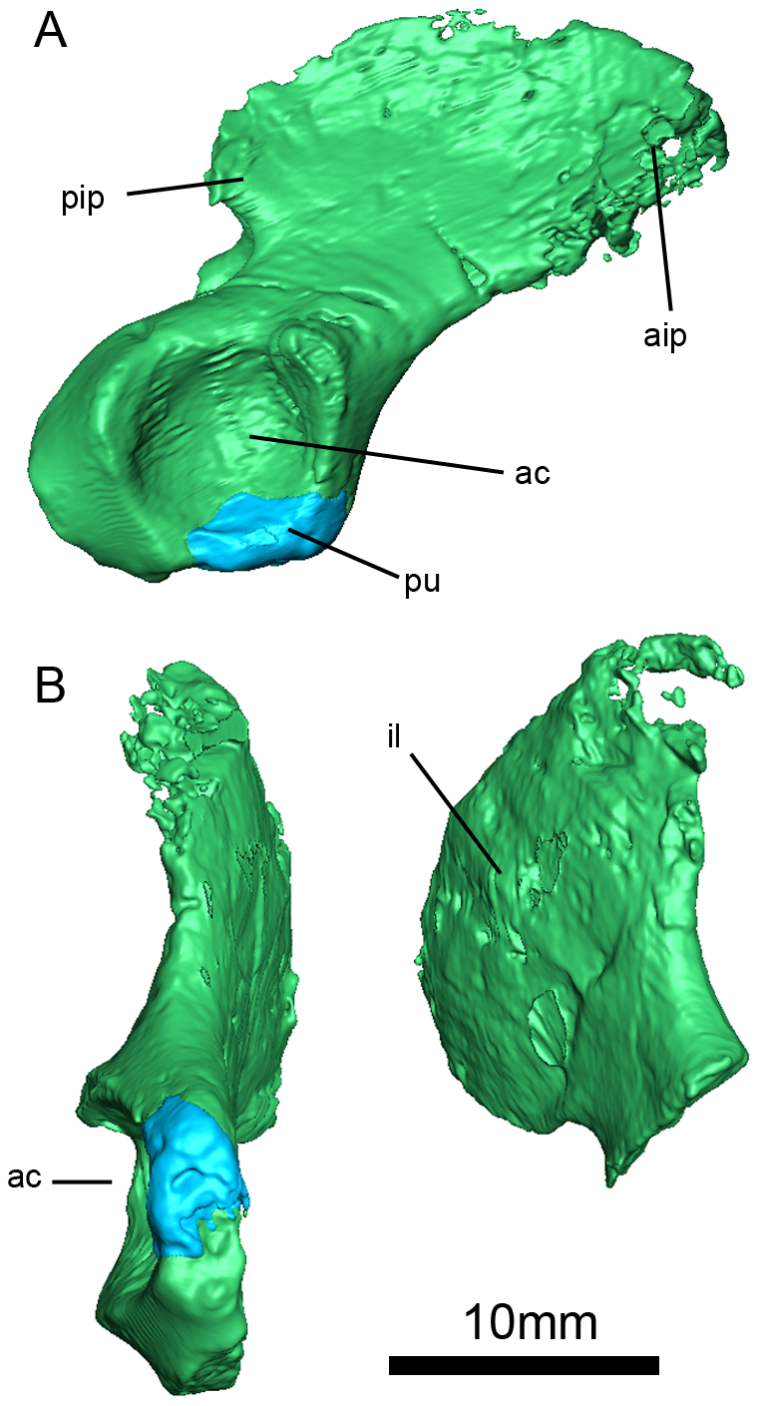
*Niassodon mfumukasi* pelvic girdle. Pelvic girdle in right lateral (A) and ventral (B) views. **ac**, acetabulum; **aip**, anterior iliac process; **il**, ilium **pip**, posterior iliac process; **pu**, pubis. Ilium and ischium and in green.

**Figure 14:**
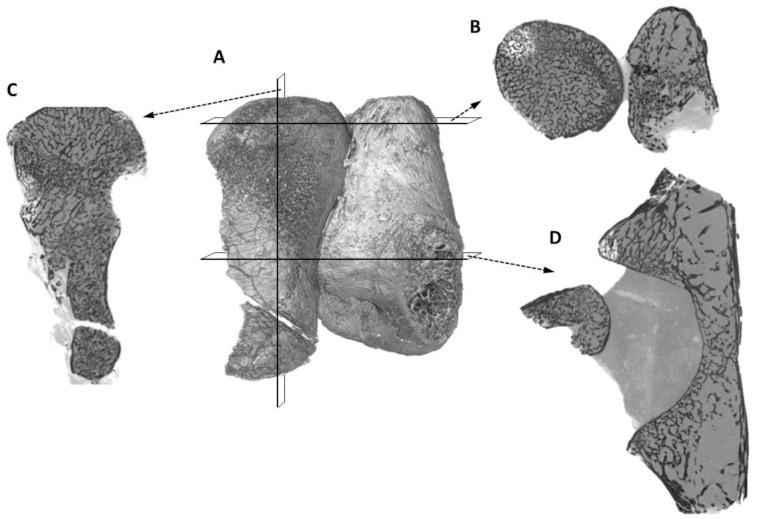
SRµCT virtual histological sectionsof the femur and partial pelvic girdle of *Niassodon mfumukasi*. (A) Volume rendering of the femur and partial pelvic girdle; (B) Proximal epiphyseal section of the femur; (C) longitudinal section along the femur; (D) diaphyseal section of the femur and acetabulum (note that the femur is not in anatomical position, but as it was found in the fossil, i.e., the femur epiphysis is dislodged from the acetabulum).

**Figure 15:**
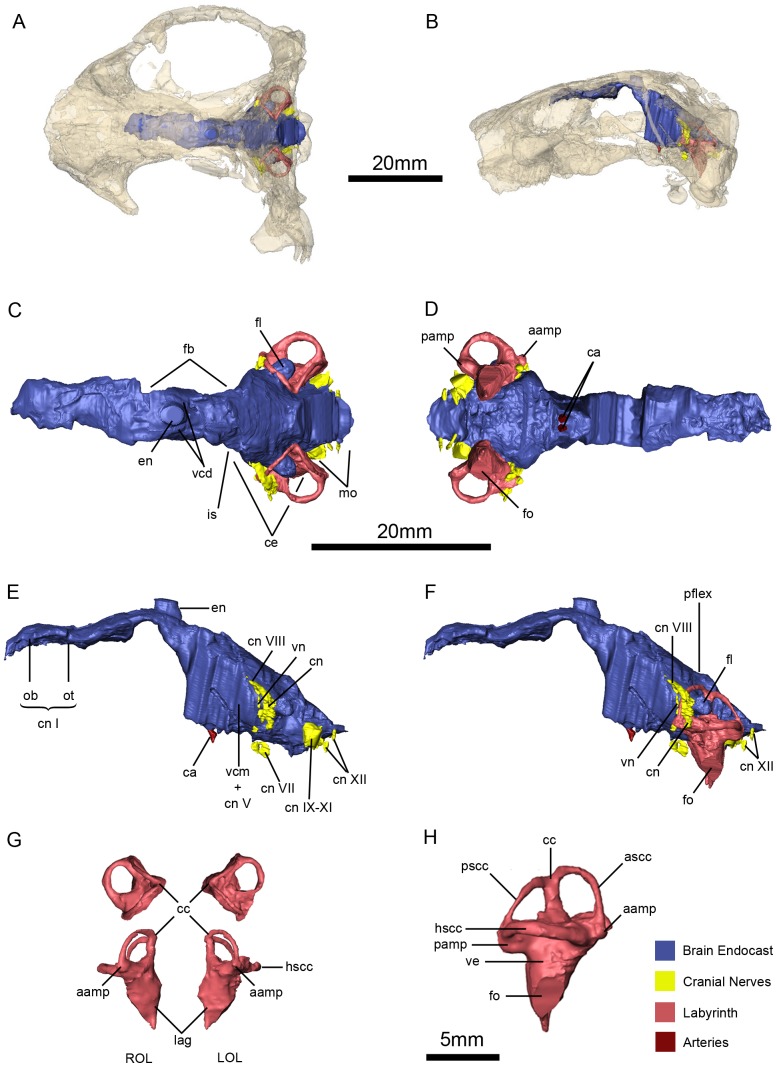
*Niassodon mfumukasi* neuroanatomy and inner ear anatomy. Cranial endocasts in the cranium in dorsal (A), and left lateral (B) views. Cranial endocasts in dorsal (C), ventral (D), left lateral without the osseous labyrinth (E), left lateral view with the osseous labyrinth (F). Both osseous labyrinths in dorsal and anterior views (G) and both osseous labyrinths in right lateral view (H). **aamp**, ampulla of the anterior semicircular canal; **ascc**, anterior semicircular canal; **ca**, carotid arteries; **cc**, crus comunis; **ce**, cerebelum; **cn**, cochlear nerve; **cn** **I,** olfactory nerve; **cn** **VII**, facial nerve; **cn** **VIII**, vestibulocochlear nerve; **cn** **IX-XI**, glossopharyngeal and vagoaccessory nerves; **cn** **XII**, hypoglossal nerves; **en**, epiphyseal nerve; **fb**, forebrain; **fl**, paraflocculus; **fo**, fenestra oval; **is**, isthmus; **hscc**, horizontal semicircular canal; **lag**, lagena; **LOL**, left osseous labyrinth; **mo**, medula oblongata; **ob**, olfactory bulb; **ot**, olfactory tract; **pamp**, ampulla of the posterior semicircular canal; **pflex**, pontine flexure **pscc**, posterior semicircular canal; **ROL**, right osseous labyrinth; **vcd**, vena capitis dorsalis; **vcm** + **cn** **V**, vena capitis medialis and trigeminal nerve; **ve**, vestibule; **vn**, vestibular nerve.

## References

[1] Castanhinha R, Araújo R, Júnior LC, Angielczyk KD, Martins GG, et al (2013) Bringing Dicynodonts Back to Life:Paleobiology and Anatomy of a New Emydopoid Genus from the Upper Permian ofMozambique. PLoS ONE 8(12): e80974 doi:10.1371/journal.pone.0080974 24324653 10.1371/journal.pone.0080974 PMC3852158

