# Supplementary figures and images for: Bringing Dicynodonts Back to Life: Paleobiology and Anatomy of a New Emydopoid Genus from the Upper Permian of Mozambique
Source: PLoS One. 2013 Dec 4;8(12):e80974. doi: 10.1371/journal.pone.0080974 (PMC3852158; doi:10.1371/journal.pone.0080974)

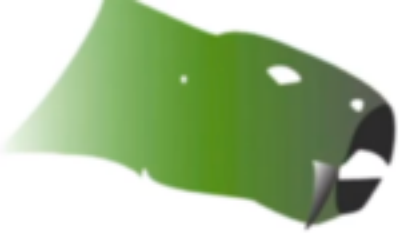

Supplement: Figure S1 — 3D interactive visualization of all preserved bones and internal structures including brain, internal ears, cranial nerves and vasculature of Niassodon mfumukasi (ML1620). (PDF) [file pone.0080974.s001.pdf]
